# Single-particle surface-enhanced coherent anti–Stokes Raman scattering: Nanoparticle design and mechanism

**DOI:** 10.1126/sciadv.ady0545

**Published:** 2026-01-28

**Authors:** Sanjun Fan, Ran Cheng, Haonan Lin, Zhewen Luo, Abigail E. Smith, Chih-Feng Wang, Jinna He, Brian T. Scarpitti, Deben N. Shoup, Hannah C. Schorr, Erjun Liang, Jian Ye, Ji-Xin Cheng, Zachary D. Schultz

**Affiliations:** ^1^ Department of Chemistry and Biochemistry, The Ohio State University, Columbus, OH 43210, USA.; ^2^ Department of Chemistry, Boston University, Boston, MA 02215, USA.; ^3^ Department of Electrical and Computer Engineering and Department of Biomedical Engineering, Boston University, Boston, MA 02215, USA.; ^4^ School of Biomedical Engineering, Shanghai Jiao Tong University, Shanghai 200030, China.; ^5^ Physical Sciences Division, Pacific Northwest National Laboratory, Richland, WA 99352, USA.; ^6^ School of Electric and Mechanical Engineering, Pingdingshan University, Pingdingshan 467000, China.; ^7^ School of Physics and Laboratory of Zhongyuan Light, Zhengzhou University, Zhengzhou 450001, China.

## Abstract

Surface-enhanced coherent anti–Stokes Raman scattering (SECARS) is believed to increase the signal intensity and sensitivity by combining the localized surface plasmon resonance and coherent Raman enhancement. However, the realization of SECARS is more complex than for surface-enhanced Raman scattering (SERS) and remains challenging. Unlike SERS, the nonlinear CARS process requires the coherent interaction of three distinct fields. The interactions between the electric fields and nanoparticle (NP) morphology for single-particle SECARS remain unexplored, and the underlying mechanisms generating the SECARS signal are not fully understood. Here, 27 distinct NPs were synthesized and screened using a CARS microscope. Among them, only star-core core-satellite NPs show single-particle SECARS signals, which were affected by laser polarization, two-photon luminescence background, photoinduced heating effects, particle size, and particle morphology. The influence of NP properties on SECARS enhancement offers guidance for the design and synthesis of NPs for single-particle SECARS and opportunities in biological imaging and chemical sensing.

## Teaser

Screening diverse nanoparticle sizes and shapes identifies properties for high-speed single-particle Raman imaging probes.

## INTRODUCTION

Raman spectroscopy is widely used to probe the molecular structure and composition of materials with minimal sample preparation and is applicable to a broad range of disciplines, from material science and chemistry to biology, forensic science, and medicine. Nevertheless, the small Raman cross-section on the level of ∼10^−30^ cm^2^ sr^−1^ results in an intrinsically weak signal that can limit its utility in certain applications (*1*). Surface-enhanced Raman scattering (SERS) is a highly sensitive analytical tool that leverages the enhancement of the electromagnetic field near metal surfaces, typically gold (Au) or silver (Ag) nanoparticles (NPs), to detect and identify low concentrations of chemical and biological analytes (*2*, *3*). The electromagnetic field enhancement near the NP surface allows SERS to achieve high sensitivity, whereas achieving acquisition rates faster than ∼10 Hz in the single- or few-molecule limit is considered challenging (*4*, *5*). Surface-enhanced coherent Raman scattering (SECRS), including surface-enhanced stimulated Raman scattering (SESRS) and surface-enhanced coherent anti–Stokes Raman scattering (SECARS), offers a promising alternative to break the speed limit imposed by SERS (*6*), which can improve the imaging speed by orders of magnitude due to the introduction of coherent Raman enhancement (*7*, *8*). In addition, the plasmonic enhancement can also contribute to the greater sensitivities, enabling single-molecule SECRS detection with a pixel acquisition time of a microsecond and below (*9*–*12*).

The continuous development of diverse plasmonic nanostructures has been fueling the advancements of SECARS since its first demonstration on a flat Ag film in 1979 (*13*). Film-based plasmonic substrates are widely used to experimentally boost SECARS signals, with broad genres spanning plain Au films with (*14*) or without (*15*) Au or silicon (Si) particles, dielectric films with Au particles (*10*, *16*), nanostructured Au or Ag surfaces (*17*, *18*), plasmonic Doppler gratings (*19*), graphene with Au pyramids (*20*), graphene oxide (*21*), self-assembled silica microspheres on polymer grating samples (*22*), and glass substrates with randomly dispersed or self-assembled Au NPs (*23*–*25*) or Si-based nanoantennas (*26*). Theoretically, film-based substrates provide more flexibility for morphological modifications via sophisticated nanofabrication, which leads to highly tunable surface plasmon resonances that match with both the pump and Stokes excitation wavelengths and anti-Stokes emission wavelength, thus enabling the strongest signal enhancement (*27*–*31*). However, similar to SERS, one limitation of film-based substrate enhancements lies in their reliance on near-field effects, which only amplify signals from analytes located in extremely close proximity to the plasmonic surface. This requirement for intimate contact restricts their applicability in biological contexts, such as bioimaging. For example, cellular imaging has been enhanced with a thin Au film, but the cells were dried prior to imaging so that the cellular components could be close to the Au surface (*32*), which hinders its potential for studying living cells or micron-thick biopsies.

Plasmonic NPs open opportunities for SECARS to be applied for bioimaging because they can be targeted to distinct locations in biological samples. By functionalizing the plasmonic NPs with Raman-active indicators, SECARS nanotags are encoded by the indicators and present bright SECARS signals for detection. When combining SECARS probes with protein-based stabilizers or targeting motifs, SECARS has broadened its biomedical applications to high-speed cellular imaging (*33*) and examining pathological biopsy via immunohistochemistry (*34*). Current applications of SECARS nanotags have used Au nanospheres, relying on simple dimerization (*9*, *35*, *36*) or passive clustering in cells or tissue (*33*, *34*) to create “hotspots” that generate bright SECARS signals. The electromagnetic enhancement factor (EF) for SECARS is theorized to be higher than that for SERS due to its higher-order dependence on incidence power and enhancement from anti-Stokes photons (*37*). However, in reality, the generation of SECARS signal from a single plasmonic NP is more challenging than SERS due to (i) the involvement of light at three distinct wavelengths (i.e., pump, Stokes, and anti-Stokes); (ii) acquisition times for SECARS that are three orders or more shorter than SERS; (iii) the generation of nonresonant background from processes like two-photon luminescence (TPL) or four-wave mixing (FWM) background from Au NPs (*25*, *38*), dependent on different excitation conditions; (iv) experimental EFs several orders of magnitude lower than expected theoretical EFs observed in clustered plasmonic nanostructures (*23*, *24*, *39*); and (v) coherent interference between fields originating within a plasmonic junction (*9*). Some of these factors can be addressed, for example, the nonresonant background can be suppressed using time-resolved surface-enhanced coherent anti–Stokes Raman scattering (tr-SECARS) spectroscopy (*24*) or wide-field FWM microscope (*40*, *41*). However, many of these complications remain puzzling. Despite the design and optimization of plasmonic NPs for single-particle SECARS performance, only Au nanodumbbells consisting of a Au NP dimer inside a silica shell have been explored for single-particle SECARS (*9*, *35*, *36*), and plasmonic NPs with other morphologies have not been successfully reported.

**Fig. 1.**
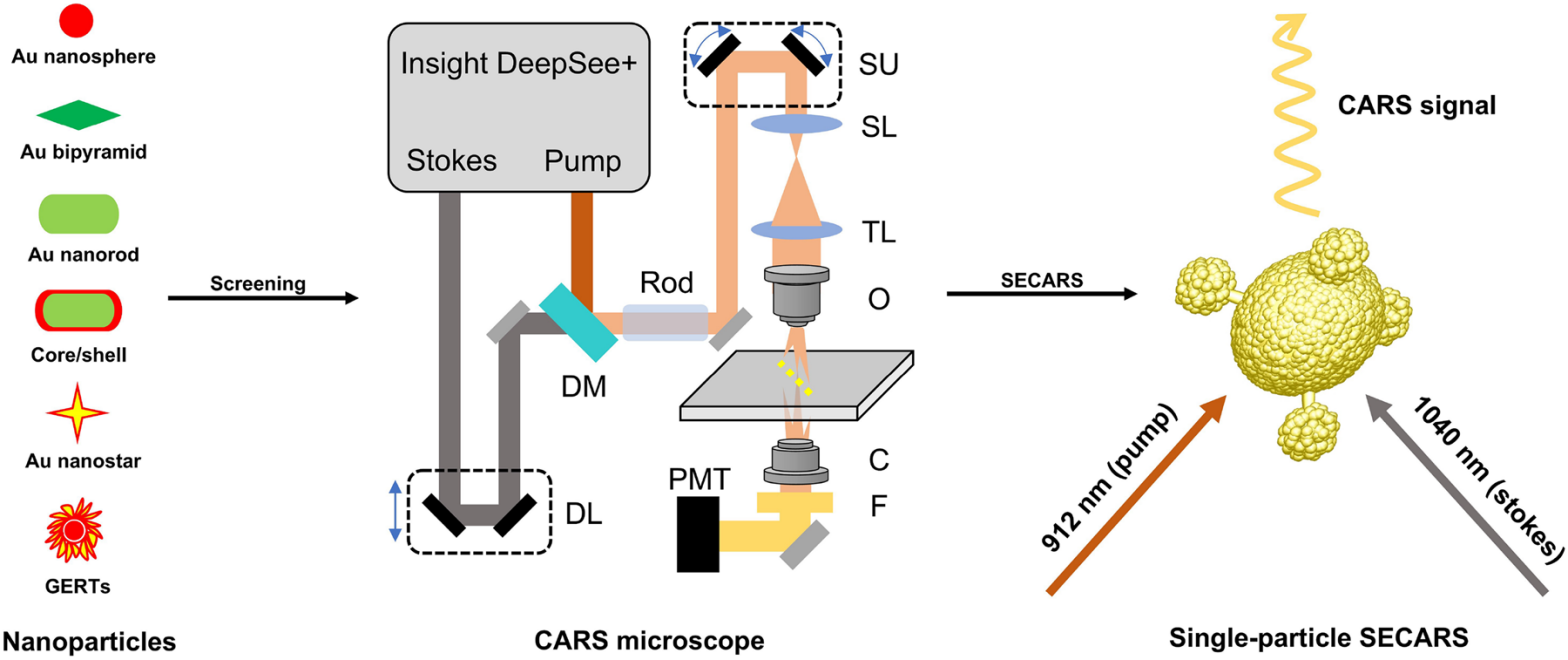
Schematic diagram for single-particle SECARS measurement. A variety of NPs are screened using a CARS microscope, and then, single-particle SECARS signal is investigated using CS NPs. C, condenser; DL, delay line; DM, dichroic mirror; F, filter; O, objective; PMT, photomultiplier tube; SL, scan lens; SU, scanning unit; TL, tube lens.

As shown in Fig. 1, we synthesized diverse shapes of NPs, including spheres, rods, stars, bipyramids, gap-enhanced Raman tags (GERTs), intragap or porous NPs, dimers, core/shell particles, and core-satellite (CS) particles. We then screened these NPs using a hyperspectral CARS microscope to quickly acquire the average SECARS signals over a large region, from which we identified the NPs with SECARS signals. Next, we performed single-particle SECARS examinations on numerous individual NPs, whose single-particle identities were confirmed via colocalized scanning electron microscopy (SEM) (*25*). We found among all the NPs screened that only star-core CS NPs showed single-particle SECARS signals. Through the study of different star-core CS NPs, experimental results showed that single-particle SECARS signal was greatly affected by laser polarization, TPL background, photoinduced heating effects, and the plasmonic properties of star-core CS NPs such as the number of hotspots, sizes, shapes, and aggregates. We further compared SERS and SECARS at the single-particle level with simulations to validate our experimental results. The results indicate that certain NPs, such as Au nanospheres, may fail to produce detectable SECARS signals due to lower overall enhancement, whereas CS NPs exhibiting the strongest electromagnetic field enhancement are detected by SECARS.

## RESULTS

### NP preparation

**Fig. 2.**
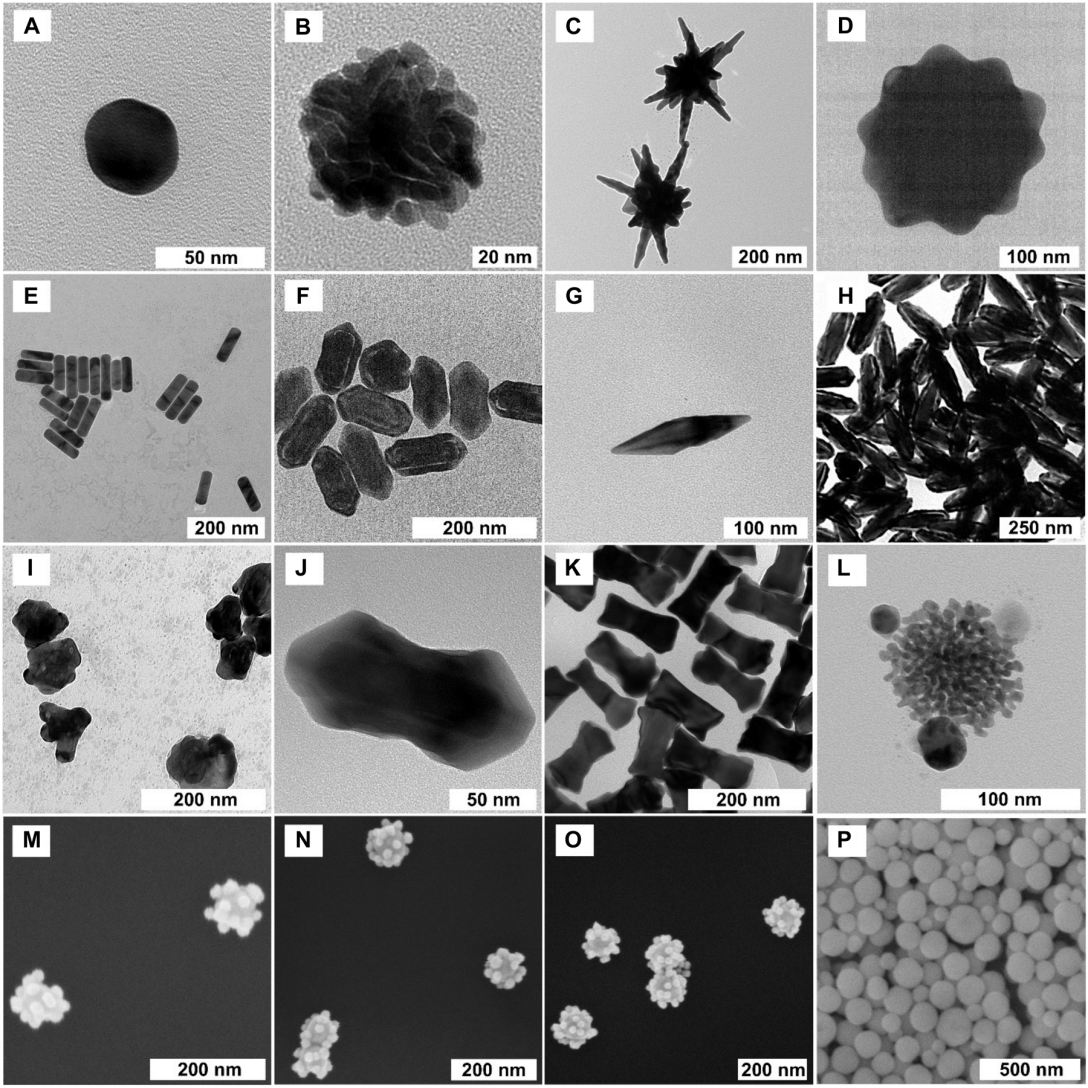
Characterization of NPs. Transmission electron microscopy (TEM) images of (**A**) Au nanosphere, (**B**) GERTs (200 μl of 10 mM HAuCl_4_), (**C**) anisotropic nanostars (20 μl of Au seed solution), (**D**) isotropic nanostar, (**E**) nanorods (0.6 ml of 10 mM HAuCl_4_), (**F**) nanorod-based intragap NPs, (**G**) bipyramid (0.5 ml of seed solution), (**H**) bipyramid-based intragap NPs, (**I**) nanostar-based porous NPs, (**J**) core/shell NPs (via method 1 in the Supplementary Materials), (**K**) core/shell NPs via method 3 in the Supplementary Materials, and (**L**) porous Au star (core)/sphere (satellite) NP; SEM images of (**M**) 50-nm sphere (core)/20-nm sphere (satellite) NPs, (**N**) 70-nm sphere (core)/20-nm sphere (satellite) NPs, (**O**) 90-nm sphere (core)/20-nm sphere (satellite) NPs, and (**P**) silica shell–coated 70-nm sphere (core)/20-nm sphere (satellite) NPs.

We synthesized a variety of NPs shown in Fig. 2 and then screened them using a CARS microscope to quickly evaluate their SECARS signals in a 40,000-μm^2^ region. These NPs include Au nanospheres, isotropic/anisotropic nanostars, nanorods, bipyramids, nanorod/bipyramid-based intragap NPs, porous NPs, core/shell NPs, GERTs, and sphere/star-core CS NPs both with and without silica shell coatings, etc. To find optimal NPs that exhibit CARS signals from single NPs, we designed the localized surface plasmon resonance (LSPR) peaks to vary broadly across the visible and near-infrared (NIR) spectral range. The LSPR peaks of diverse NPs at 543 nm (GERTs), 544 nm (nanosphere), 578 nm (isotropic nanostars), 540 and 628 nm (core/shell), 517 and 723 nm (bipyramids), 581 and 863 nm (bigger core/shell), 520 and 874 nm (porous CS NPs), 820 and 931 nm (anisotropic nanostars), 506 and 1028 nm (nanorods), etc. were measured as shown in figs. S1 to S3. In addition, we synthesized silica shell–coated sphere-core CS NPs (sphere-core SiO_2_-CS NPs) using a linker [11-mercaptoundecanoic acid (11-MUA) paired with polyethylenimine (PEI)], because this configuration creates nanogaps, or hotspots, between the core and satellites, which is believed to intensify the electromagnetic fields. First, as shown in fig. S4, we synthesized several different sizes of nanospheres, among which the nanospheres (50/70/90 nm) were used as cores and the nanospheres (12 and 20 nm) served as satellites. Second, two kinds of CS NPs [(50-12, 70-12, 90-12 nm) and (50-20, 70-20, 90-20 nm)] were then prepared as shown in fig. S5 and Fig. 2 (M to O), and their corresponding extinction spectra were shown in fig. S5. The size distribution of spherical and silica shell–coated CS NPs was shown in fig. S6 (G and H). With increased sizes of cores and satellites, the LSPR peaks exhibited a red shift. For example, the silica shell–coated CS NPs in fig. S6 (D to F) show that the major peak at 678 nm red-shifts to 713 nm when the core size increases. Last, sphere-core SiO_2_-CS NPs were obtained in Fig. 2P and fig. S6 with varied LSPR peaks within the region of 630 to 720 nm.

### SECARS signal screening

**Fig. 3.**
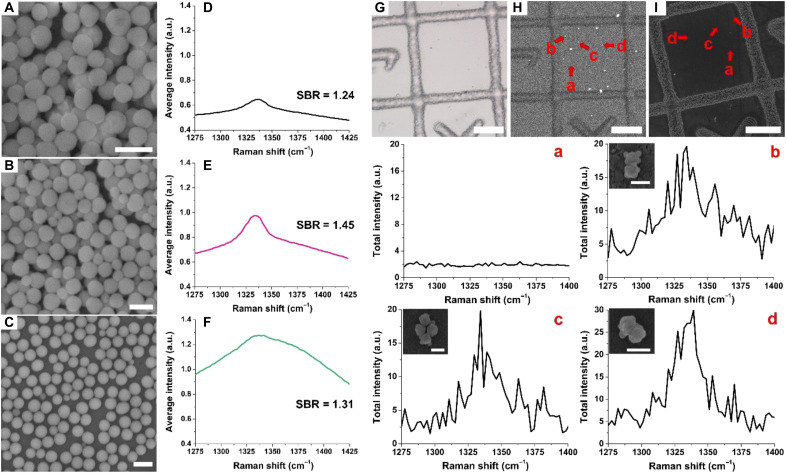
SECARS signals of sphere-core SiO_2_-CS NPs. Ensemble-averaged SECARS signals of sphere-core SiO_2_-CS NPs (**A**) 50-20 nm, (**B**) 70-20 nm, and (**C**) 90-20 nm, measured across three distinct sample regions. Scale bars, 200 nm. Colocalized SECARS and SEM studies guided by a labeled glass substrate. (**D**) SECARS signal of NPs in (A). (**E**) SECARS signal of NPs in (B). (**F**) SECARS signal of NPs in (C). The signal to background ratio (SBR) is noted for (D to F). (**G**) A bright-field image of the photoetched glass substrate with labeled grids. The unlabeled grid in the center was defined as the ROI. Scale bar, 20 μm. (**H**) The averaged SECARS image of a hyperspectral imaging stack that covered the ROI. Scale bar, 20 μm. (**I**) SEM image of the same ROI. Scale bar, 20 μm. Below are SECARS spectra of the bare glass substrate without nanotag presented from location a, and nanotags presented at locations b to d. The corresponding nanostructures were shown by SEM images as the upper left insets. Scale bars, 200 nm. SECARS spectra (a to d) were collected at 600 × 600 pixels per frame with 60 sequential frames, a pixel size of 150 nm, and a pixel dwell time of 10 μs per pixel per frame and power set at 200 μW for both pump and Stokes beams. The intensities are noted in arbitrary units (a.u.).

We first screened 20 types (Fig. 2, A to L and P, and figs. S1F, S2, D and H, S3, and S6, A and D to F) out of 27 NPs prepared the NP preparation section under a CARS microscope to quickly evaluate their CARS signals by averaging the overall signals generated by NPs over the 40,000-μm^2^ field of view. As shown in fig. S7, out of the 20 types of NPs with different morphologies, only bipyramid-based intragap NPs (fig. S7H), nanostar-based porous NPs (fig. S7I), and sphere-core SiO_2_-CS NPs (Fig. 3, A to C) exhibited weak ensemble SECARS signals. However, no SECARS signal can be detected from them at the single-particle level, which suggests that the SECARS we observed from ensemble samples incorporates some level of interparticle coupling rather than from intrinsic single-particle enhancement (*42*). Regarding the sphere-core SiO_2_-CS NPs, we found that the ensemble SECARS signals from 50-20 nm sphere-core SiO_2_-CS NPs was higher than 50-12 nm sphere-core SiO_2_-CS NPs (fig. S8), which was ascribed to both larger satellite sizes used and a main, red-shifted CS coupling LSPR peak at 678 nm of 50-20 nm sphere-core SiO_2_-CS NPs. In addition, a new shoulder peak at 547 nm was observed for 50-20 nm sphere-core SiO_2_-CS NPs due to interparticle coupling among satellites (fig. S6), contributing to the overall stronger plasmonic enhancement. We further explored whether increasing the size of cores could also help with the improvements of SECARS performances. We observed that as the core sizes increased, the TPL background rose substantially for the 70-20 nm and 90-20 nm sphere-core SiO_2_-CS NPs (Fig. 3, D to F), most likely due to enhanced radiative damping, which commonly occurs in larger or resonant structures (*43*, *44*). The intensity ratios of the SECARS peak and the background increased when changing the core size from 50 to 70 nm but substantially decreased when core size increased to 90 nm. Based on these results, 70-20 nm sphere-core SiO_2_-CS NPs were selected as the optimal candidate for single-particle SECARS examinations due to the highest signal-to-background ratio observed in the ensemble sample.

To perform the single-particle SECARS study, highly diluted 70-20 nm sphere-core SiO_2_-CS NPs were drop-casted and dried on the photoetched glass substrate with 50-μm labeled grids as shown in Fig. 3G, where the labels served to identify the region of interest (ROI). SECARS hyperspectral imaging was first performed, by which SECARS spectra from nanostructures were obtained and NP distributions were simultaneously revealed by the average image of the whole hyperspectral imaging stack (Fig. 3H). SEM imaging was performed after SECARS on the exact same area (Fig. 3I). Notably, the SEM image and the SECARS image are mirror-symmetric to each other, as revealed by the photoetched labels. The SECARS spectrum of the bare glass substrate without nanotags present was measured at location a, and we observed a weak background without any distinct peak present. We also observed SECARS signals originating from b or d individual NPs when they were in close vicinity, as shown at location b and c or from an NP dimer wrapped in the same silica shell as shown at location d. Notably, there were no distinct SECARS signals observed from single NPs using silica shell–coated spherical CS NPs. However, these silica-coated particles provide important information to guide the design of NPs where star cores were used for single-particle SECARS.

### Star-core CS NP synthesis, SECARS measurement, and simulations

**Fig. 4.**
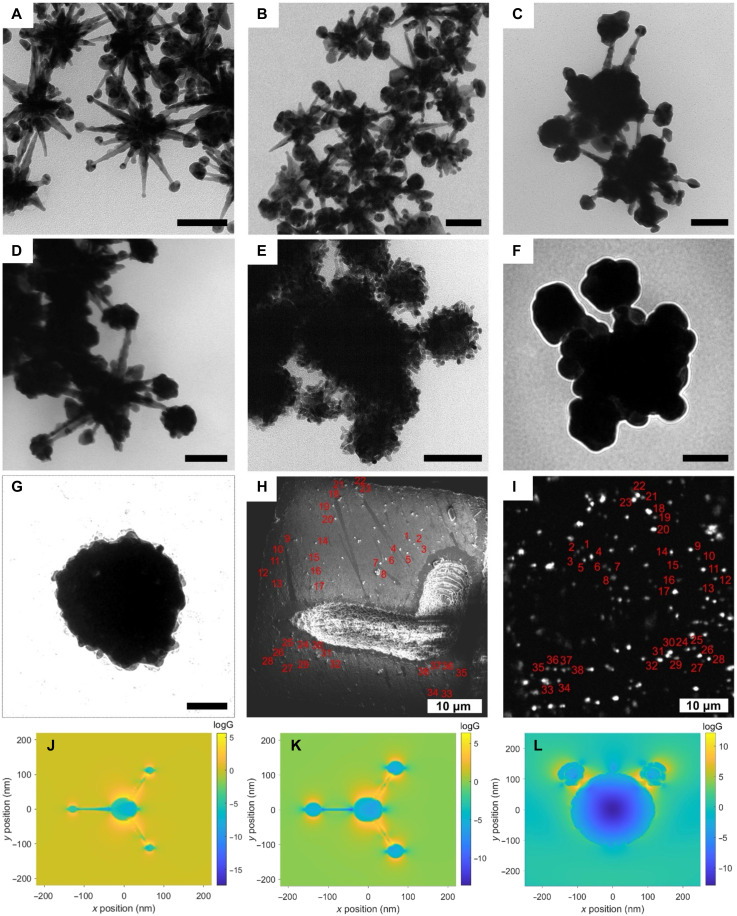
Star-core CS NP synthesis, SECARS measurement, and simulations. Star-core CS NP synthesized using a linker-free method. (**A**) CS NPs (8 ml of seed solution), (**B**) CS NPs (4 ml of seed solution), (**C**) CS NPs (2 ml of seed solution), (**D**) CS NPs (1 ml of seed solution), (**E**) CS NPs (0.5 ml of seed solution), (**F**) CS NPs (0.25 ml of seed solution), and (**G**) CS NPs (0.125 ml of seed solution). Scale bars, 100 nm. (**H**) SEM image of letter “L” on the photoetched glass substrate with labeled grids, on which CS NPs [0.25 ml of seed solution; NP in (F)] spread. (**I**) The averaged SECARS image of a hyperspectral imaging stack that covered the same ROI of (H). The total EFs [*G* = *g*_p_^4^*g*_s_^2^*g*_as_^2^; *g*_p_, *g*_s_, and *g*_as_: EF at the wavelengths of pumping (912 nm), Stokes (1040 nm), and anti-Stokes (812 nm)] are presented for (**J**) CS NP [4 ml of seed solution; NP in (B)], (**K**) CS NP [2 ml of seed solution; NP in (C)], and (**L**) asymmetric CS NP [0.25 ml of seed solution; NP in (F)].

Considering that the CS NP is a promising structure for single-particle SECARS, we therefore modified the morphology to further red-shift the LSPR and introduce more and stronger hotspots to enhance the SECARS signal. A linker-free method was developed to synthesize star-core CS NPs (*45*), avoiding the usage of linkers such as PEI or DNA. As shown in Fig. 4 (A to G), the satellite spheres continuously grow bigger and transform to GERTs containing plenty of gaps (Fig. 4E) with increased ratio between Au^3+^ and nanostar seeds added during the satellite growth step. The star-core CS NP last grew into a big sphere (0.125-ml seed solution) shown in Fig. 4G. The NPs (Fig. 4F) that preserved the nanostar core-GERTs satellite structures covered by a thin Au shell layer were used for single-particle SECARS experiments. To understand the factors that contribute to the generation of strong SECARS signals from single star-core CS NPs, the electromagnetic fields excited at 633 nm from NPs with different morphologies (Fig. 4, B to G) in solution and their corresponding extinction spectra were calculated through finite-difference time-domain (FDTD) simulations in fig. S9 (A to E). As shown in fig. S9 (A to D), as the sizes of satellites increased and the morphologies of satellites grew from spheres to GERTs, the enhancement of the electromagnetic field grew stronger. Last, the EF of the CS NPs (Fig. 4F) reaches the highest value. However, when the CS NPs grew into a spherical structure (fig. S9E), the electromagnetic field dropped compared to the CS NPs (Fig. 4F), which indicated that the CS structure is a preferred structure for generating SECARS signal. In addition, as shown in Fig. 4, the simulated SECARS electromagnetic field EFs of CS NPs (Fig. 4, B, C, and F) on glass are 10^5^ (Fig. 4J), 10^6^ (Fig. 4K), and 10^13^ (Fig. 4I), respectively. Although FDTD simulations predict SECARS field enhancement on the order of 10^13^ for the particle in Fig. 4F, the experimentally derived EF (Supplementary Materials, calculation of experimental enhancement factors section) is lower (*25*). This discrepancy is consistent with known coherence effects, where the experimental SECARS signal decreases due to destructive interference arising from phase mismatch between the optical fields in the sample (*9*). These challenges are overcome in the CS NPs (Fig. 4F), enabling single-particle SECARS from this nanostructure.

Matching the pump and Stokes excitation wavelengths as well as the anti-Stokes emission wavelength to the LSPR contributes to CS NPs (Fig. 4F) generating the strongest SECARS signal. The pump beam and the anti-Stokes beam are reported to play a decisive role in the enhancement from a plasmonic Doppler grating (*19*). As shown in fig. S9 (A to D and F), the main band in the extinction spectrum of the star-core CS NPs red-shifts from 534 nm (Fig. 4B) to 630 nm (Fig. 4F) with the increase of satellite size, and the second peak at longer wavelengths (972 to 886 nm) appears more notable. Furthermore, as shown in fig. S10, FDTD simulation of the field enhancement of three electromagnetic fields corresponding to pump, Stokes, and anti-Stokes (anti-Stokes: 812 nm, pump: 912 nm, and Stokes: 1040 nm) shows a higher degree of overlap of hotspots achieved on CS NPs (Fig. 4F), compared to the CS NPs (Fig. 4C). Achieving the spatial overlap of hotspots between the tips of nanostar core and satellite at the wavelengths of pump, Stokes, and anti-Stokes is desirable to further intensify SECARS signals, as previously confirmed by studying Fano-resonance plasmonic structures (*46*, *47*).

To facilitate the occurrence of single particles, NP suspensions were diluted more than 20×, drop-casted, and dried on a clean photoetched glass substrate with labeled grids. Hyperspectral SECARS imaging was performed on a randomly selected ROI (letter “L”), by which SECARS spectra from nanostructures were obtained and NP distributions were simultaneously revealed by the average image of the whole hyperspectral imaging stack (Fig. 4I). SEM imaging was then performed after SECARS on the exact same ROI (Fig. 4H). As similarly observed in Fig. 3, the SEM image and the SECARS image were mirror-symmetric to each other, revealed by the pattern of NP distributions (Fig. 4, H and I).

### Single-particle CARS of star-core CS NP

**Fig. 5.**
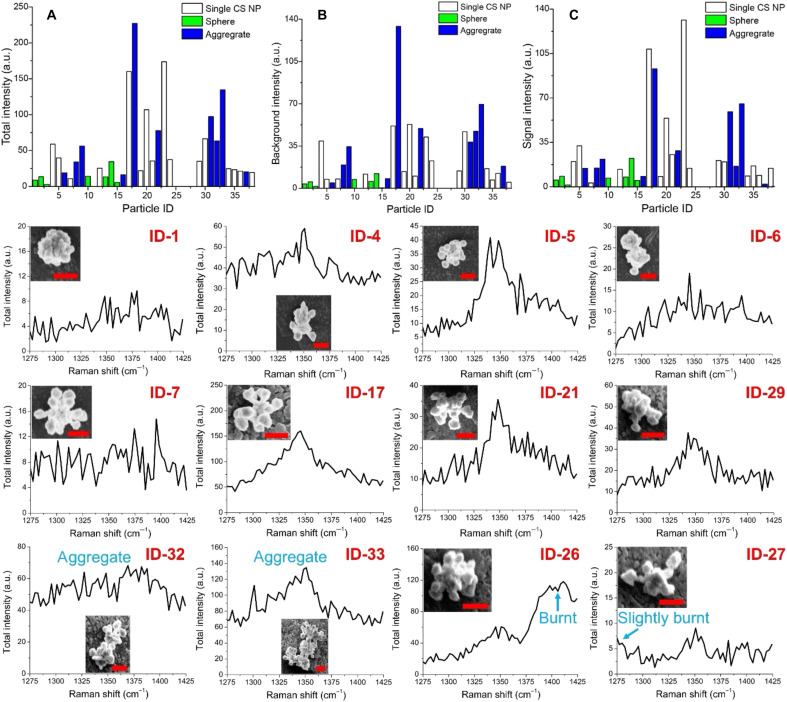
Single-particle SECARS of CS NPs. (**A**) Total intensity of NPs, (**B**) background intensity of NPs, and (**C**) signal intensity (total intensity − background intensity) of NPs shown in Fig. 4F; NPs 11 and 25 to 28 exhibited the signs of photodamaging and are therefore excluded in (A) to (C). SECARS signals from single NPs and aggregates are shown below. Their particle IDs correspond to the numbers in Fig. 4 (H or I). Scale bars, 200 nm. SECARS spectra were extracted from the hyperspectral imaging stack consisting of 60 sequential frames of 300 × 300 pixels per frame with a pixel size set at 200 nm and the power of both beams (pump and Stokes) set at 250 μW with a dwell time of 10 μs per pixel.

Colocalization studies with SECARS and SEM enabled the SECARS spectra of NPs, NP total intensity (the peak intensity of SECARS spectrum × pixels), NP TPL background intensity (the average intensity of the first five points of the SECARS spectrum), and SECARS signal intensity (total intensity – background intensity) to be correlated with NP morphologies. The intensity overview (total intensity, background intensity, and signal intensity) of NPs with different morphologies including single star-core CS NPs, single spheres, and aggregates was analyzed and presented in Fig. 5 (A to C). Compared to the star-core CS NPs (Fig. 4F), we did not find any SECARS signal from CS NPs with small spherical satellites (Fig. 4, A and B) or spherical NPs (Fig. 4G). As the satellite sizes increase (Fig. 4, C to E), SECARS signals of CS NPs are rarely observed for the CS NPs shown in Fig. 4C but are observed for a portion of CS NPs shown in Fig. 4 (D and E). For example, although some NPs (Fig. 4D) exhibit CARS signals (fig. S11), their intensities are not as high as that of NPs with more gaps (Fig. 4F). These results further support previous findings that plasmonic films or aggregated NPs with better resonance matching to the LSPR and a better spatial overlap of hotspots at excitation and emission wavelengths yield stronger SECARS signals.

The detailed single-particle SECARS spectra covering the Raman shift region associated with the ν(NO_2_) (peak at ∼1340 cm^−1^) of the 4-nitrobenzenethiol (4-NBT) absorbate are shown in Fig. 5. Specifically, no SECARS signal from single spherical NPs (ID-1) was observed. Depending on the morphologies and spatial hotspot distribution, NPs-ID-5, 17, 21, and 29 exhibited high SECARS signals, NP-ID-4 showed low SECARS signals, whereas no or very weak signal from CS NPs-ID-7 was observed. Notably, the TPL background of aggregates (ID-32 and 33) was observed to be higher than most of the single CS NPs. It should also be noted that it is difficult to confirm that the SECARS signal from the aggregates is higher than single CS NPs based on our experimental observations. This phenomenon is different from SERS, where aggregating particles generally increase signals. Occasionally, we observed thermal damage of NPs (ID-26 and 27) due to the photoinduced heating effects, which was indicated by an acute intensity increase shown in the SECARS spectrum. As shown in fig. S12, the diverse extinction spectra (absorbance and dark-field scattering) from single CS NP varied with slight alterations of the CS NP morphology, leading to different degrees of LSPR matching with the wavelengths of pump, Stokes, and anti-Stokes, and thus contributing to different SECARS signals observed experimentally as shown in Fig. 5. Of further importance for single-particle detection, the single-particle extinction spectrum is typically narrower than the ensemble extinction spectrum.

### Polarization analysis

**Fig. 6.**
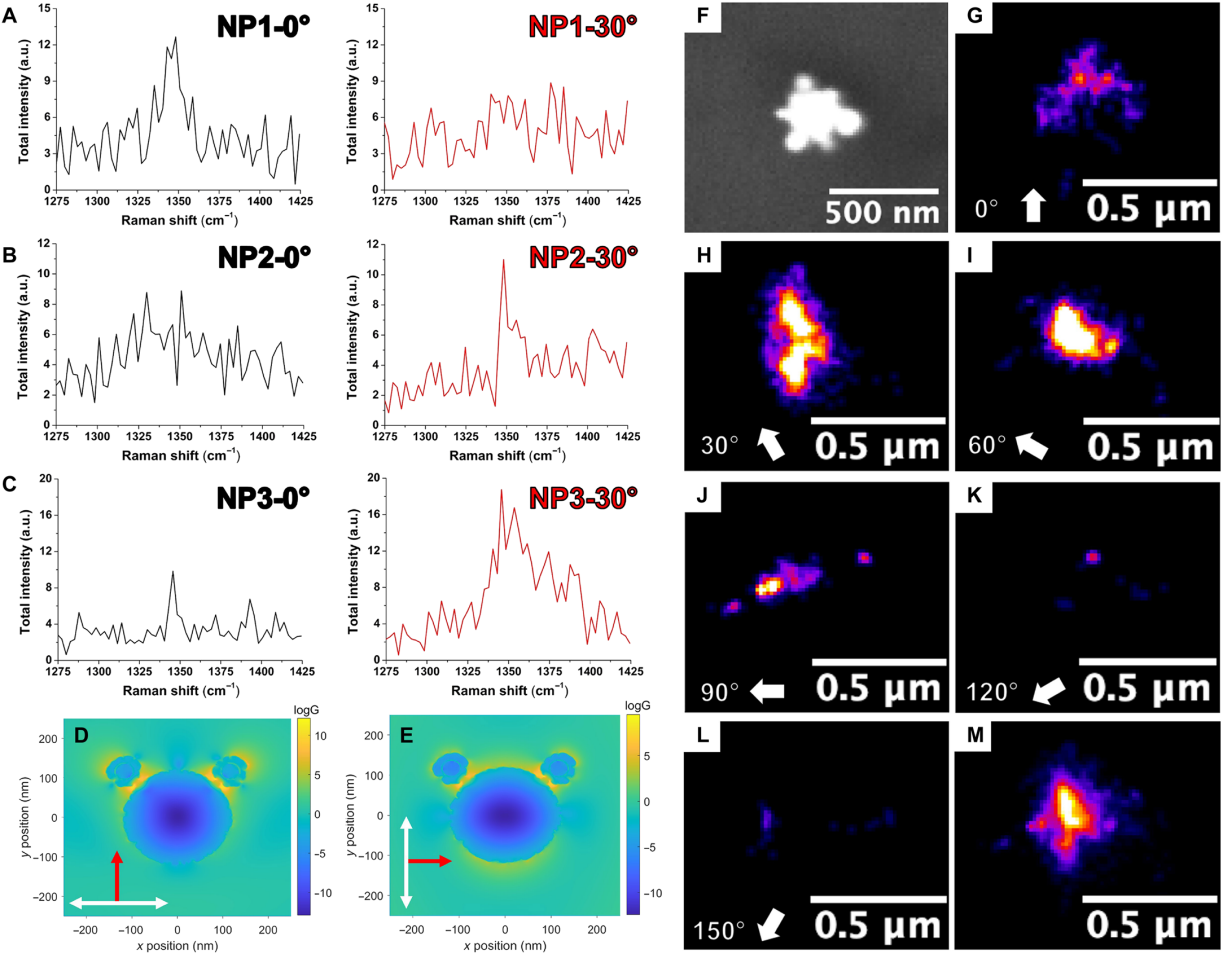
Polarization dependence of SECARS and SERS. (**A** to **C**) The polarization dependence of CARS signals of three NPs by changing the polarization by 30°. The polarizations of the pump (912 nm) and Stokes (1040 nm) beams were simultaneously varied. The total EFs [*G* = *g*_p_^4^*g*_s_^2^*g*_as_^2^; *g*_p_, *g*_s_, *g*_as_: EF at the wavelengths of pumping (912 nm), Stokes (1040 nm), and anti-Stokes (812 nm)] are presented for (**D**) asymmetric CS NP (Fig. 4F; EF = 10^13^) and (**E**) asymmetric CS NP (Fig. 4F; polarization direction changes 90°, EF = 10^9^). The white arrows represent the magnetic field component, whereas the red arrows indicate the electric field component. (**F**) SEM image of single CS NP, and its super-resolution spectral images with HAWK preprocessing at different angles [0° (**G**), 30° (**H**), 60° (**I**), 90° (**J**), 120° (**K**), and 150° (**L**)]. The arrows indicate the polarization direction of the incident light. (**M**) Overlay of all polarizations.

Despite the best SECARS performance being observed from the star-core CS NPs shown in Fig. 4F, we observed variation between the single-particle SECARS signals in Fig. 5, which could be ascribed to the varied degree of alignment between CS NP hotspots, laser polarization, and the nonuniformity of CS NP morphologies. In the FDTD simulation (fig. S10), the hotspots of CS NPs (Fig. 4F) are located between the satellites and the tips of nanostar core. However, the hotspots of the CS NPs were not spatially distributed in a uniform format. Therefore, when examined by our linearly polarized CARS microscope, the degree of alignment between the CS NP hotspots and polarization direction of excitation fields varied across different CS NPs randomly distributed on the glass substrate. To confirm this hypothesis, we investigated the influence of the polarization direction on the SECARS signals by using the same glass substrate with randomly distributed CS NPs and simultaneously rotating the incident linear polarizations of both lasers by 30°. As shown in Fig. 6 (A to C), the SECARS spectra of CS NPs exhibited three different phenomena: (i) disappearance (NP1), (ii) appearance (NP2), and (iii) enhancement (NP3). Simulation results further confirmed that changing the polarization direction can substantially alter the SECARS EF of star-core CS NP from 10^13^ (Fig. 6D) to 10^9^ (Fig. 6E), thus greatly altering the SECARS signals generated. This discrepancy is consistent with coherence-related effects, particularly the dependence of the SECARS signal on phase-aligned molecular polarizations induced by the pump and Stokes fields. Aligning the incident fields with high-field enhancement aspects is also reported to be important in SERS (*48*), and the polarization results with SECARS suggest similar, perhaps greater dependence. We also investigated the influence of polarization direction on the SERS signals. As shown in Fig. 6 (G to M), Haar wavelet kernel (HAWK) preprocessed super-resolution spectral images of single star-core CS NP were shown with varied polarization from 0° to 150° at a step of 30°. Despite the polarization changes, SERS signals were consistently observed at each polarization angle from the single particle (fig. S13), which was different from that observed in SECARS where single-particle SECARS could fully disappear when linear polarization changed by only 30° (Fig. 6A). Second, the nonuniformity of star-core CS NP morphologies could also contribute to the variations of SECARS signals, for example, asymmetric CS NP has a higher EF (fig. S14A; 10^13^) than that of the symmetric CS NP (fig. S14B; 10^12^).

### Single-particle SERS

**Fig. 7.**
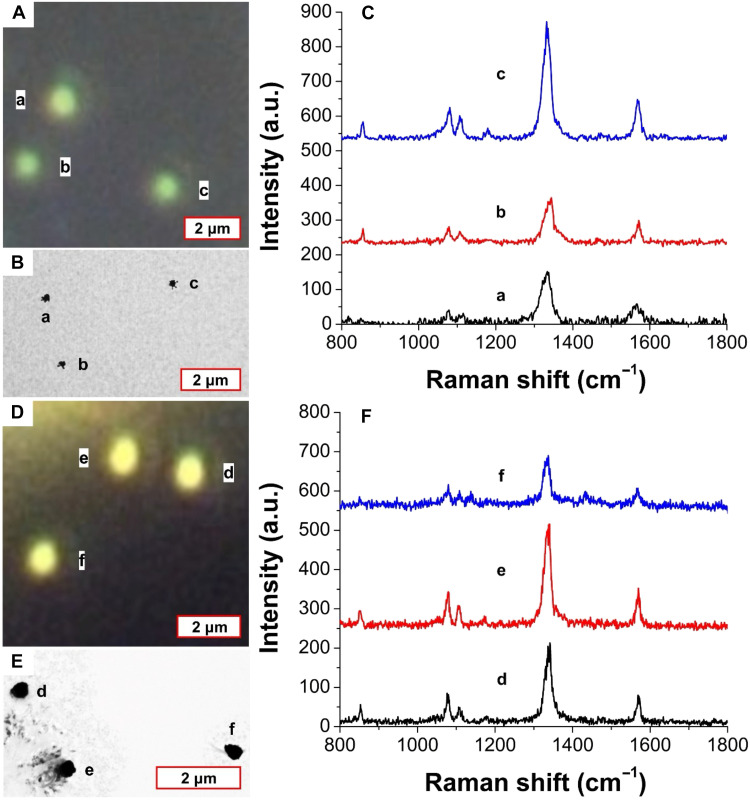
Single-particle SERS. (**A**) Dark-field microscopy of star-core CS NPs (Fig. 4E). (**B**) TEM image of NPs in (A). (**C**) Raman spectra of the NPs in (A). (**D**) Dark-field microscopy of spheres (Fig. 4G). (**E**) TEM image of the NPs in (D). (**F**) Raman spectra collected using 1-s acquisition from the NPs in (D). The Raman spectra shown were recorded with 1-s acquisition times and a 633-nm laser with 0.22 mW at the sample.

We further compared SECARS and SERS at the single-particle level using CS NPs synthesized with different morphologies. To perform single-particle SERS, these CS NPs were first diluted and then drop-casted on the transmission electron microscopy (TEM) grids. During and after SERS measurements, dark-field microscopy (Fig. 7, A and D) and TEM (Fig. 7, B and E) were used to correlate the NPs with their single-particle SERS performance, respectively. As discussed above, only a portion of CS NPs (e.g., the structure shown in Fig. 4E) showed SECARS signals, and the larger CS particles with spherical structure (ID-1 shown in Fig. 5) showed no SECARS signals. However, we easily observed SERS signals from each individual NP (Fig. 7, C and F). Although it appears that the SERS measurement generates higher signals than the SECARS measurement for single particles, the result is slightly misleading. The spontaneous Raman measurement is a substantially longer acquisition, limited by the readout time of the array detector. In this case, we used 1 s per pixel acquisition times. Calculating the signal-to-noise ratios (SNRs) for the single-particle SERS signals in Fig. 7, we see an SNR range from 20 to 60. Approximating the SNR scales with the square root of the difference in acquisition time, and using the 10-μs dwell time from the SECARS measurement and averaging 60 frames (as reported in our results), none of these particles are detectable by SERS at SNR > 2 with this faster acquisition rate. SECARS is providing a larger enhancement in our experiments.

Our SECARS and SERS comparison study suggests that although aggregation can create hotspots that strengthen the enhancement of SERS and SECARS at the single-particle level, achieving SECARS signal is more difficult than SERS. Their underlying physical mechanisms and sensitivities to aggregation differ markedly. This difference may arise from phase relationships and interference but may also reflect the absolute enhancements to the individual fields driving the increased signals as well as the polarization dependence, which affects both SERS and SECARS.

## DISCUSSION

SECARS is widely regarded as a promising approach for substantially enhancing both signal intensity and detection sensitivity. However, in practice, particularly at the single-particle level, these expectations are not achieved. Here, we screened a diverse array of NPs for particles that showed promise in SECARS imaging at rapid acquisition rates (10-μs dwell time). We screened 27 types of NPs with different morphologies: sphere, rod, star, bipyramid, GERTs, intragap or porous NPs, dimers, core/shell, and CS via CARS microscope to evaluate their SECARS performances. CS NPs (Fig. 4F) showed the best SECARS signals at the single-particle level. The failure of these other particles reflects the common perception that SECARS does not work as expected. This shortfall is largely attributed to the inherent complexity of the SECARS process, which involves a nonlinear optical mechanism and stringent phase-matching requirements. These factors remain poorly understood at the single-particle scale, presenting challenges to the reliable implementation and interpretation of SECARS experiments. In our work we observed multiple factors that notably affected the observed signals, including laser polarization, TPL background, photoinduced heating effects, the plasmonic properties of CS NPs such as the number of hotspots, sizes, shapes, and aggregates, and spatial overlap of hotspots at pump, Stokes, and anti-Stokes wavelength. Although hotspots can strengthen both the enhancement of SERS and SECARS signals at the single-particle level, there appears to be differences in the dependence on the resulting signal that are attributed to the nonlinear optical process, including phase relationships and the extent to which each field is enhanced by the plasmon resonance, which fundamentally distinguishes it from linear techniques such as SERS. Specifically, the geometries within NP aggregates and in different configurations of CS NPs appear to disrupt the phase relations and local coherence required for efficient SECARS signal generation. The complex interaction of surface plasmons and light at multiple distinct wavelengths (pump, Stokes, and probe beams) for generating a coherent signal at the anti-Stokes frequency within a much shorter acquisition time over nonresonant background imposes more demanding requirements on NP structural design. Nonetheless, by successfully distinguishing a single-particle geometry that produces SECARS, this work helps to elucidate the relationship between the design of plasmonic NPs and SECARS signal generation, providing guidance on SECARS nanoprobe designs and opening opportunities for the broad application in SECARS-based ultrafast bioimaging and biosensing.

## MATERIALS AND METHODS

### Materials and chemicals

Trisodium citrate dihydrate (C_6_H_9_Na_3_O_9_, ≥99%), silver nitrate (AgNO_3_, ≥99%), sodium borohydride (NaBH_4_, 99%), l-ascorbic acid (AA, ≥99%), hydrochloric acid (HCl, 37%), ammonium hydroxide solution (NH_4_OH, 28%), hydrogen tetrachloroaurate(III) hydrate (HAuCl_4_·3H_2_O, 99.995%), tetraethoxysilane (TEOS, >99.999%), polyvinylpyrrolidone (PVP, molecular weight of ~55,000), PEI (>99.5%), ethanol (ACS reagent, ≥99.5%), isopropanol (IPA, ≥99.9%), diethylene glycol (DEG, ≥99%), *N*,*N*-dimethylformamide (DMF, ≥99.8%), 11-MUA (>95%), sodium hypochlorite solution (NaClO, 10 to 15%), dimethylamine (DMA, 40% in H_2_O), cetyltrimethylammonium chloride solution (CTAC, 25 wt % in H_2_O), and hexadecyltrimethylammonium bromide (CTAB, ≥99%) were purchased from Sigma-Aldrich. 4-NBT (96%) was purchased from Thermo Fisher Scientific. TEM grids (CF150-Cu and FFLF135-Cu-UL) were purchased from Electron Microscopy Sciences. The glassware was cleaned by piranha solution and sonicated in ethanol for 30 min prior to use. Ultrapure water (18.2 MΩ cm) was used in all the experiments.

### Nanoparticle preparation

#### Au nanosphere preparation

Au nanosphere preparation has been reported in our previous work with a slight change (*49*). Specifically, Au seeds were prepared by combining 5 ml of 0.5 mM HAuCl_4_ and 5 ml of 200 mM CTAC into a 20-ml glass vial under vigorous stirring at 1100 rpm. After stirring for 1 min, 0.6 ml of chilled, freshly made 10 mM NaBH_4_ was added to reduce Au^3+^ to ~3-nm Au seeds, used below as the seed solution. The seed solution was then left undisturbed to age for at least 3 hours. Au sphere cores (10 nm) were synthesized from 40 ml of 100 mM CTAC, 30 ml of 100 mM AA, and 40 ml of 0.5 mM HAuCl_4_ added into 250-ml round-bottom flask under vigorous stirring at 1100 rpm, followed by the addition of 800 μl of seed solution. After 30 min of stirring, ~10-nm Au spheres were obtained. The Au spheres were increased in size using a growth solution consisting of 40 ml of 100 mM CTAC, 0.3 ml of 100 mM AA, and 2 ml of the 10-nm Au sphere solution combined into 100-ml round-bottom flask under vigorous stirring at 1100 rpm. After stirring for 2 min, 10 ml of 2 mM HAuCl_4_ solution was added dropwise to the flask using a syringe pump at the speed of 5 ml/hour (*50*). The extinction of Au sphere is shown in fig. S1. To improve homogeneity, 10 ml of the above Au NP solution was added into a 15-ml centrifuge tube, and then, 320 μl of 1.0 to 1.5% NaClO and 210 μl of 5 mM HAuCl_4_ were added simultaneously. This tube was then shaken for 30 min and was stored overnight at room temperature before use. This step ensures round spheres with little particle-to-particle variation. The Au nanospheres were incubated with excess 4-NBT overnight at room temperature and were then washed with water by centrifugation (20 min at 10,000 rpm) for characterization.

#### Anisotropic Au nanostar preparation

Anisotropic Au nanostars were prepared using a seed-mediated growth method, as described in the previous work (*51*). Specifically, 50 ml of 1 mM HAuCl_4_ was prepared in a 125-ml Erlenmeyer flask and boiled for 10 min before 7.5 ml of 1% (w/v) trisodium citrate was added to the solution. The seed solution was stirred for 15 min at 700 rpm while kept boiling then for another 15 min without heating to cool. Next, in a 100-ml round-bottom flask, 30 ml of ultrapure water was stirred at 1100 rpm, and 200 μl of 60 mM HAuCl_4_, 40 μl of 1 M HCl, and 20 μl or 80 μl seed solution were added (fig. S2, A to C). After stirring for 2 min, 80 μl of 10 mM AgNO_3_ and 200 μl of 100 mM AA were added. The anisotropic Au nanostars were synthesized after the solution color turned black. The anisotropic Au nanostars were incubated with excess 4-nitrothiophenol overnight at room temperature and were then washed with water by centrifugation (20 min at 8000 rpm) for characterization.

#### Symmetric Au nanostar preparation

Symmetric nanostars were prepared according to previous literature with a slight change (*52*). First, icosahedral Au seeds were prepared by combining 0.1 g of PVP and 25 ml of DEG into a 100-ml round-bottom flask under heating until refluxing. After 5 min, 2 ml of DEG containing 20 mg of HAuCl_4_·3H_2_O was quickly added into the flask. After a 10-min reaction, the reaction was stopped and cooled to room temperature. The product was washed with DMF twice by centrifugation. Last, the icosahedral Au seeds were dispersed in 27 ml of DMF. Second, 1.2 g of PVP was dissolved in 15 ml of DMF, and then, 100 μl of DMA (40%) and 400 μl of 2.5 M HCl were added. Subsequently, 1 ml of Au icosahedral seed solution was added, followed by the addition of 166 μl of 60 mM HAuCl_4_ solution. Last, the reaction solution was gently stirred in an oil bath at 90°C for 4 hours. The product was centrifuged and washed with ethanol twice and lastly dispersed in 2 ml of water. The symmetric Au nanostars were incubated with excess 4-nitrothiophenol overnight at room temperature and were then washed with water by centrifugation (20 min at 8000 rpm) for characterization.

#### Au nanorods and Au nanorod-based intragap NP preparation

Au nanorods were prepared using a modified method based on the literature reported by Wang’s group (*53*). Specifically, 5 ml of 0.5 mM HAuCl_4_ and 5 ml of 200 mM CTAB were added into a 20-ml glass vial under vigorous stirring at 1200 rpm. After 1 min, 0.6 ml of chilled, freshly made 10 mM NaBH_4_ was quickly added to reduce Au^3+^ to ~3-nm Au seeds (brown color), which was used as the seed solution. The seed solution was then left to age undisturbed for at least 3 hours. For nanorod preparation, 19 ml of 100 mM CTAB, 1.8 ml or 0.6 ml of 10 mM HAuCl_4_, and 0.6 ml of 10 mM AgNO_3_ were added in sequence to a 100-ml round-bottom flask, and the solution became yellow under stirring at 1200 rpm. Subsequently, 1 ml of 100 mM hydroquinone was then added into the flask, and the color quickly disappeared. Last, this flask was kept in a 30°C water bath overnight after adding 150 μl of seed solution as prepared above. The Au nanorods were incubated with excess 4-nitrothiophenol overnight at room temperature and were then washed with water by centrifugation (20 min at 8000 rpm) for characterization. The preparation of Au nanorod-based intragap NP has been reported in our previous work (*54*). Specifically, 16 ml of 50 mM CTAB, 2 ml of Au nanorod solution, and 200 μl of 2 mM 4-nitrothiophenol were added in sequence to a 100-ml round-bottom flask. After stirring for 10 min at 1200 rpm, 200 μl of 10 mM AgNO_3_ was added into this flask, followed by the addition of 20 μl of 10% NH_4_OH. Subsequently, after 5 min, 200 μl of 100 mM AA, 200 μl of 10 mM HAuCl_4_ and 200 μl of 1 M HCl were added in sequence to this flask. The prepared solution eventually turned orange after overnight incubation in 60°C water bath, and then, the NPs were centrifuged out (10 min at 4000 rpm) for characterization.

#### Au bipyramids and Au bipyramid-based intragap NP preparation

The synthesis of Au bipyramids was reported previously by Wang’s group (*55*). Briefly, 10 ml of 100 mM CTAC, 1 ml of 5 mM HAuCl_4_, and 1 ml of 0.1 M trisodium citrate were added into 9 ml of Milli-Q water in a 20-ml glass vial while stirring vigorously at 1200 rpm. This was followed by the addition of 500 μl of chilled, freshly made 25 mM NaBH_4_ solution. After stirring for 2 min, this vial was heated to 80°C for 6 hours until the color changed from brown to shiny red. The resultant solution was used as seed solution. Two hundred milliliters of 0.1 M CTAB, 10 ml of 10 mM HAuCl_4_, 2 ml of 10 mM AgNO_3_, and 4 ml of 1 M HCl were added in sequence to a 500-ml Erlenmeyer flask, and the solution became yellow under stirring at 1200 rpm. A total of 1.6 ml of 0.1 M AA was then added into the flask, and the color quickly disappeared. Last, this flask was kept in a 30°C water bath overnight after adding 500 μl or 6 ml of seed solution as prepared above. The Au bipyramids were incubated with excess 4-nitrothiophenol overnight at room temperature and were then washed with water by centrifugation (20 min at 8000 rpm) for characterization. The preparation steps of Au bipyramid-based intragap NPs are similar to Au nanorod-based intragap NPs (*54*). Specifically, 16 ml of 50 mM CTAB, 2 ml of the Au bipyramid solution, and 200 μl of 2 mM 4-nitrothiophenol were added in sequence to a 100-ml round-bottom flask under stirring at 1200 rpm. After 10 min, 200 μl of 10 mM AgNO_3_ was added into this flask, followed by the addition of 50 μl of 10% NH_4_OH. Subsequently, after 5 min, 200 μl of 100 mM AA, 200 μl of 10 mM HAuCl_4_, and 100 μl of 1 M HCl were added in sequence to this flask. Last, the prepared solution eventually turned gray/purple after overnight incubation in 60°C water bath, and then, these NPs were centrifuged out (10 min at 3500 rpm) for characterization.

#### Au nanostar-based porous Au NP preparation

There were two steps to synthesize porous nanostar NPs. First, small Au nanostars were prepared according to the previous work (*56*). Specifically, 100 ml of 1 mM HAuCl_4_ was prepared in a 125-ml Erlenmeyer flask and boiled before 15 ml of 1% (w/v) trisodium citrate was added to the solution. The seed solution was stirred for 15 min at 700 rpm while kept boiling and then for another 15 min without heating to cool. Next, in a 100-ml round-bottom flask, 19 ml of ultrapure water was stirred at 1100 rpm, and 1 ml of 5 mM HAuCl_4_, 200 μl of 1 M HCl, and 400 μl of seed solution were added. After stirring for 2 min, 60 μl of 10 mM AgNO_3_ and 100 μl of 100 mM AA were added. The anisotropic Au nanostars were synthesized after the solution color turned black, followed by the addition of 2 ml of 100 mM CTAB. Second, 16 ml of 50 mM CTAB, 200 μl of 2 mM 4-nitrothiophenol, and 2 ml of anisotropic Au nanostar solution were added into 100-ml round-bottom flask. After a 30-min incubation, 100 μl of 10 mM AgNO_3_, 20 μl of 10% NH_3_·H_2_O, and 200 μl of 100 mM AA were added into flask in sequence. After 10 min, 200 μl of 10 mM HAuCl_4_ and 200 μl of 1 M HCl were added. Last, the prepared solution was stored in 60°C water bath overnight, and then, these NPs were centrifuged out (20 min at 8000 rpm) for characterization.

#### Au nanorod-based core/shell NP preparation

There are three methods to make Au nanorod-based core/shell NPs: (Method 1) 16 ml of 50 mM CTAB, 2 ml of Au nanorod solution, and 200 μl of 2 mM 4-nitrothiophenol were added in sequence to a 100-ml round-bottom flask. After stirring for 10 min at 1200 rpm, 200 μl of 10 mM AgNO_3_ was added into this flask, followed by the addition of 20 μl of 10% NH_4_OH. Subsequently, after 40 min, 200 μl of 100 mM AA and 400 μl of 10 mM HAuCl_4_ were added in sequence to this flask. The prepared solution was stored in 60°C water bath overnight, and then, the NPs were centrifuged out (20 min at 8000 rpm) for characterization. (Method 2) 16 ml of 50 mM CTAB, 2 ml of Au nanorod solution, and 200 μl of 2 mM 4-nitrothiophenol were added in sequence to a 100-ml round-bottom flask. After stirring for 10 min at 1200 rpm, 100 μl of 10 mM AgNO_3_ was added into this flask, followed by the addition of 30 μl of 10% NH_4_OH. Subsequently, after 5 min, 200 μl of 100 mM AA, 200 μl of 10 mM HAuCl_4_, and 200 μl of 1 M HCl were added in sequence to this flask. The prepared solution was stored in 60°C water bath overnight, and then, the NPs were centrifuged out (20 min at 8000 rpm) for characterization. (Method 3) 16 ml of 50 mM CTAB, 200 μl of 2 mM 4-nitrothiophenol, and 2 ml of Au nanorod solution were added into 100-ml round-bottom flask. After a 30-min incubation, 100 μl of 10 mM AgNO_3_, 400 μl of 10 mM HAuCl_4_, 200 μl of 100 mM AA, and 50 μl of 1 M HCl were added. Last, the prepared solution was stored in 60°C water bath overnight, and then, these NPs were centrifuged out (20 min at 8000 rpm) for characterization.

#### Au bipyramid-based core/shell NP preparation

For the preparation of Au bipyramid-based core/shell NPs, 16 ml of 50 mM CTAB, 2 ml of the Au bipyramid solution, and 200 μl of 2 mM 4-nitrothiophenol were added in sequence to a 100-ml round-bottom flask under stirring at 1200 rpm. After 30 min, 200 μl of 100 mM AA and 200 μl of 10 mM HAuCl_4_ were added in sequence to this flask. Last, the NPs were synthesized after overnight incubation in 60°C water bath, and then, these NPs were centrifuged out (10 min at 3500 rpm) for characterization.

#### GERT preparation

The GERT NPs are synthesized according to procedure reported by Khlebtsov *et al.* (*57*). Briefly, at first, 10-nm Au NPs were synthesized, which has been described in the Au nanosphere preparation section. Next, for petal growth to form the GERTs, 16 ml of 50 mM CTAC, 200 μl of 2 mM 4-NBT, and 1 ml of 10-nm Au NP solution were added into a 50-ml round-bottom flask in sequence. After stirring at 1100 rpm for 10 min, 200 μl or 600 μl of 10 mM HAuCl_4_ solution, and 200 μl of 100 mM AA were added. This reaction took 30 min under vigorous stirring, and then, the NPs were centrifuged out (20 min at 8000 rpm) for characterization.

#### Silica shell–coated Au sphere-core CS NPs preparation

*Au nanosphere preparation*. 12-nm Au NPs (satellites): Au NP satellites with an average diameter of 12 nm were synthesized as follows: 96 ml of deionized (DI) water was heated to 100°C in a 250-ml flask. A condenser was used to prevent the evaporation of the solvent. Four milliliters of HAuCl_4_ solution (25 mM) was added to the flask under vigorous stirring. Once the boiling had commenced again, 15 ml of sodium citrate solution (1 wt %) was quickly added. The color of the solution changed from light yellow to pinkish red within 5 min. The mixture was kept boiling for 30 min.

20-nm Au NPs (satellites): Spherical Au NP cores with diverse sizes were synthesized by following a multistep seeded growth synthetic protocol developed by Bastús *et al.* with modifications (*58*). This protocol was adopted due to the high size tunability, high size uniformity, and high particle density of the synthesized Au NPs. First, the seed solution was synthesized as follows: 141.5 ml of DI water was heated to 100°C in a 250-ml flask. A condenser was used to prevent the evaporation of the solvent. One milliliter of HAuCl_4_ solution (25 mM) was added to the flask under vigorous stirring. Once the boiling had commenced again, 8.5 ml of sodium citrate solution (1 wt %) was quickly added. The color of the solution changed from light yellow to pink within 5 min. The mixture was kept boiling for 30 min to form Au NPs with an average diameter of around 20 nm, which were used as the seeds for the multistep Au NP synthesis below or as satellites.

30-nm Au NPs: After the synthesis of the seeds (20 nm), the continuing size growth of Au NPs was conducted at 90°C. The above seed solution was cooled down to 90°C, and then, 50 ml of seed solution was extracted to maintain a volume of 100 ml in the flask. Then, 0.67 ml of HAuCl_4_ solution (25 mM) was quickly added, and the mixture was kept at 90°C under vigorous stirring. After 30 min, another 0.67 ml of HAuCl_4_ solution (25 mM) was quickly added again, and the mixture was kept at 90°C under constant vigorous stirring for another 30 min. The average size of Au NPs in the flask was about 30 nm after these two steps.

50-nm Au NPs (cores): The above 30-nm Au NPs were used as the new seeds in the synthesis of Au NPs with larger sizes. The above 30-nm Au NP solution was diluted by extracting 50 ml of NP solution and then adding 50 ml of DI water and 3 ml of sodium citrate solution (1 wt %). The system was then stabilized for 15 min to allow the temperature to recover to 90°C. Then, 1 ml of HAuCl_4_ solution (25 mM) was quickly added, and the mixture was kept at 90°C under vigorous stirring for 30 min. This procedure was then repeated for another two times, resulting in the formation of 50-nm Au NPs.

70-nm Au NPs (cores): The above 50-nm Au NPs were used as the new seeds in the synthesis of Au NPs with larger sizes. The above 50-nm Au NP solution was diluted by extracting 50 ml of NP solution and then adding 50 ml of DI water and 3 ml of sodium citrate solution (1 wt %). The system was then stabilized for 15 min to allow the temperature to recover to 90°C. Then, 1 ml of HAuCl_4_ solution (25 mM) was quickly added, and the mixture was kept at 90°C under vigorous stirring for 30 min. This procedure was then repeated for another two times, resulting in the formation of 70-nm Au NPs.

90-nm Au NPs (cores): The above 70-nm Au NPs were used as the new seeds in the synthesis of Au NPs with larger sizes. The above 70-nm Au NP solution was diluted by extracting 50 ml of NP solution and then adding 50 ml of DI water and 3 ml of sodium citrate solution (1 wt %). The system was then stabilized for 15 min to allow the temperature to recover to 90°C. Then, 1 ml of HAuCl_4_ solution (25 mM) was quickly added, and the mixture was kept at 90°C under vigorous stirring for 30 min. This procedure was then repeated for another two times, resulting in the formation of 90-nm Au NPs.

*Modification of Au NP cores with PEI*. The synthesis of CS NPs was achieved by following a synthetic protocol developed by Pazos-Perez *et al.* with modifications (*59*), where the assembly was achieved by electrostatic absorption between positively charged, PEI-functionalized cores and negatively charged, citrate-stabilized satellites. Two milliliters of the above Au NP cores (50-, 70-, and 90-nm cores) was diluted five times by adding 8 ml of DI water in a 50-ml centrifuge tube. A mixture containing 0.15 ml of 11-MUA solution (0.1 mM 11-MUA in ethanol) and 0.04 ml of ammonia solution (2.9% in DI water) was added to the diluted Au NP core solution drop by drop during vigorous vortex and was kept under vortex for 30 min to allow sufficient 11-MUA functionalization of the cores (Au NP cores@11-MUA). Then, 0.3 ml of 4-NBT (1 mM 4-NBT in DI water, diluted from 5 mM 4-NBT solution in ethanol) was rapidly added to the mixed solution to encode Au NP cores with 4-NBT Raman indicators and kept under vigorous vortex for 1 hour. The solution was then kept undisturbed overnight at room temperature for cofunctionalizing Au NP cores with 4-NBT and 11-MUA (Au NP cores@11-MUA/NBT). On the second day, the Au NP cores@11-MUA/NBT were washed twice with DI water by centrifugation at 800*g* (for 50- and 70-nm cores) or 500*g* (for 90-nm cores) for 15 min, recovered to a total volume of 2 ml and then slowly added drop by drop under vigorous vortex to a separately prepared PEI solution made by 8 ml of PEI aqueous solution (4 mg/ml, previously sonicated for over 30 min) diluted by 8 ml of DI water. The vortexing was continued for 2 hours for PEI wrapping on Au NP cores to obtain Au NP cores@11-MUA-PEI/NBT. The PEI-functionalized cores were washed three times with DI water by centrifugation at 800*g* (for 50- and 70-nm cores) or 500*g* (for 90-nm cores) for 15 min to thoroughly remove the excess PEI to avoid the formation of chain-like structures protruding from the core particles after satellite assembly. Last, the Au NP cores@11-MUA-PEI/NBT were dispersed in DI water to reach a total volume of 2 ml for satellite assembly in the next step. Notably, no sonication was applied in all processes to avoid the disruption of the polyelectrolyte layer on the cores.

*Assembly of sphere-core CS NPs*. Since Au NPs (12- or 20-nm satellites) were stabilized by sodium citrate, they naturally carried negative surface charges. Therefore, no further surface functionalization was conducted on the satellites. Basically, 0.75 ml of Au NP cores@11-MUA-PEI/4-NBT was added drop by drop under vigorous vortex to 12 ml of above-synthesized 12-nm Au NP satellites or 24 ml of above-synthesized 20-nm Au NP satellites. The mixture was vigorously vortexed for at least 4 hours and then was shook on a shaker at the maximum speed overnight at room temperature. The CS NPs were purified with DI water three times by centrifugation at 500*g* to 600*g* (for 50- and 70-nm cores) or 300*g* to 400*g* (for 90-nm cores) for 10 min and then recovered to 1 ml with DI water.

*Synthesis of sphere-core SiO_2_-CS NPs*. Sphere-core SiO_2_-CS NPs were synthesized by absorbing the Raman indicator molecules, 4-NBT, on the surfaces of sphere-core CS NPs and coating the indicator-absorbed sphere-core CS NPs in silica shell. We adopted the synthetic protocol developed by Harmsen *et al*. (*60*). In brief, 1 ml of the above sphere-core CS NPs solution in DI water was added to a solution containing 0.15 ml of NH_4_OH (29% aqueous solution) and 7.5 ml of IPA under vigorous vortex. The silica shell growth solution containing the Raman indicator molecules was prepared separately by adding 0.1 ml of 4-NBT solution (5 mM in ethanol) and 0.15 ml of TEOS to 2.4 ml of IPA. Then, the well-mixed shell growth solution was quickly added to the basic solution containing sphere-core CS NPs described above under vigorous vortex for 1 min, and the mixture was kept undisturbed for 14 min. The reaction was paused by adding ethanol to 50 ml, followed by immediate centrifugation at 3000*g* for 10 min. The CS nanotags were then washed three times with ethanol by centrifugation at 3000*g* for 10 min and then redispersed in 1 ml of ethanol and stored at 4°C for future usage.

#### Au star-core CS NPs preparation using a linker-free method

First, Au nanostars were prepared using a seed-mediated growth method, as described in the previous work (*51*), with slight modification. Briefly, 50 ml of 1 mM HAuCl_4_ was added into a 125-ml Erlenmeyer flask and boiled for 10 min before 7.5 ml of 1% (w/v) trisodium citrate was added to the solution. The seed solution was stirred for 15 min at 700 rpm while kept boiling and then for another 15 min without heating to cool. Next, in a 100-ml round-bottom flask, 30 ml of ultrapure water was stirred at 1200 rpm, and then, 200 μl of 60 mM HAuCl_4_, 40 μl of 1 M HCl, and 80 μl of seed solution were added. After stirring for 2 min, 80 μl of 10 mM AgNO_3_ and 200 μl of 100 mM AA solution were added. The Au nanostars were synthesized after the solution color turned black. After stirring for 1 min, 1 ml of 10 mM 4-nitrothiophenol was added into the solution for 30-min incubation under stirring. Last, the Au nanostars were centrifuged out (10 min at 2000 rpm) and then resuspended in 10 ml of 50 mM CTAC. Second, in a 100-ml round-bottom flask, 16 ml of 50 mM CTAC was added. Different amounts (0.125, 0.25, 0.5, 1, 4, and 8 ml) of the Au nanostar solutions prepared above were added into the flask, respectively. Subsequently, 20 μl of 2 mM 4-nitrothiophenol, 200 μl of 10 mM HAuCl_4_, and 200 μl of 100 mM AA were added in sequence. Last, all of the solutions were put into a 37°C water bath overnight. The solution was then centrifuged (10 min at 2000 rpm) for characterization.

#### Porous Au star-core CS NPs preparation

The Au nanostar seed synthesis has been described in the Au star-core CS NPs preparation using a linker-free method section. Next, in a 100-ml round-bottom flask, 30 ml of ultrapure water was stirred at 1200 rpm, and then, 200 μl of 60 mM HAuCl_4_, 40 μl of 1 M HCl, and 80 μl of seed solution were added. After stirring for 2 min, 80 μl of 10 mM AgNO_3_ and 200 μl of 100 mM AA solution were added. The Au nanostars were synthesized after the solution color turned black. After stirring for 1 min, 1 ml of 100 mM CTAB was added. Five milliliters of Au nanostar solution, 5 ml of H_2_O, and 200 μl of 2 mM 4-nitrothiophenol were added into 50-ml round-bottom flask. After 10 min, 300 μl of 10 mM AgNO_3_, 30 μl of 100 mM AA solution, and 20 μl of 10% NH_3_·H_2_O were added into the flask. After 10 min, 200 μl of 1 M HCl and 200 μl of 10 mM HAuCl_4_ were then added into the flask. Last, the solution was put into a 37°C water bath overnight. The solution was then centrifuged (10 min at 2000 rpm) for characterization.

### Nanoparticle characterization

The synthesized NPs were characterized by SEM, TEM, and ultraviolet-visible (UV-Vis) extinction spectroscopy (VWR UV-1600PC spectrometer or Shimadzu UV-1900i spectrometer). TEM images were obtained using a Tecnai F20 or Tecnai F30 electron microscope at the Center for Electron Microscopy and Analysis (CEMAS) at The Ohio State University. The instrument was operated in STEM (scanning transmission electron microscopy) mode, and a high-angle annular dark-field detector was used for all acquisitions. For TEM characterization of NPs, carbon film–coated copper grids (CF150-Cu, Electron Microscopy Sciences) were used. For correlative TEM and Raman measurements, London finder grids (FFLF135-Cu-UL, Electron Microscopy Sciences) were used to locate Raman mapped regions in the electron microscope. SEM images were acquired using a Thermo Fisher Scientific Apreo SEM operating in Mode 2 (Optiplan) with beam energies from 5 to 10 kV and beam currents from 50 pA to 0.1 nA. The Trinity2 detector was used to detect secondary electrons. Samples were imaged as prepared with the addition of copper tape for grounding purposes. SEM images were obtained by Supra55-VP SEM (Zeiss, Boston University). For characterizations of sphere-core CS NPs and sphere-core SiO_2_-CS NPs, *NP*s were washed one to two times by centrifugation with DI water (for sphere-core CS NPs) or ethanol (for sphere-core SiO_2_-CS NPs), concentrated to 10 to 50 times of its original concentration via centrifugation and then dried on a Si wafer under vacuum at room temperature. For single-particle study, photoetched glass substrate with NPs distributed on top was first sputtered with a thin layer of Au/Pd before SEM imaging for better conductivity. SEM imaging was conducted at 3 or 5 kV with a working distance of around 6 mm and an aperture size of 10 μm. The diameters of NPs were measured by ImageJ when applicable.

### Single-particle star-core CS NP polarization measurement

#### Wide-field spectral SERS imaging

A 660-nm single-mode longitudinal diode laser (Laser Quantum) was directed through a linear polarizer (Thorlabs) and a 𝛌/2 waveplate (Edmund Optics) and then focused to a 35-μm spot size with an *f* = 25.4-mm lens (Thorlabs). The resulting scattering is collected with a 100×, 1.3 numerical aperture (NA) oil immersion objective (Olympus) and then passes through a 650-nm dichroic mirror (Thorlabs) and a 664-nm longpass filter (Semrock) prior to exiting the microscope. The signal is then directed through another 664-nm longpass filter (Semrock) and a 300 g/mm transmission diffraction grating (Thorlabs) before it is focused onto a complementary metal-oxide semiconductor detector (ORCA-Flash 4.0 V2, Hamamatsu, Ltd.). The spectral SERS images were collected with a power density of 3 kW/cm^2^ and 20-ms acquisitions. One thousand frames were taken at each polarization angle.

#### STORM and HAWK super-resolution

Image processing and analysis was done with ImageJ (National Institutes of Health), and stochastic optical reconstruction microscopy (STORM) fittings and drift corrections were done with the ThunderSTORM ImageJ plugin (*61*). An ImageJ plugin developed by the Cox group was used for HAWK image preprocessing and STORM image reconstruction as described previously (*48*, *62*).

### Single-particle dark-field scattering and absorption experiment

#### Sample preparation

The star-core CS NPs (0.5 ml of Au nanostar solution as seed) were prepared by first sonicating for 10 min. Following sonication, 10 μl of the NP solution was diluted with 150 μl of DI water. The diluted NP solution was then drop-casted onto a cleaned glass coverslip (Ted Pella, no. 260150) and left to dry overnight in a fume hood.

#### Dark-field scattering experiment

The dark-field scattering experiment was performed using a white light laser (NKT Photonics, SuperK Extreme) as the excitation source. The light was focused onto the sample at a 25° angle relative to the surface using an infinity-corrected objective (Mitutoyo, M Plan Apo NIR 100×). The scattered photons were collected by a top-mounted objective (Mitutoyo, M Plan Apo NIR 10×). To spatially filter the collected signal, a 4*f* imaging system was used, utilizing a 75-μm diameter pinhole. The filtered light was directed into a fiber-coupled spectrometer (Horiba, iHR320) connected to an electron multiplying charge-coupled device (Andor, Newton DU970P-BVF) using reflective collimators (Thorlabs, RC12SMA). The excitation power during the experiment was 100 μW, and the exposure time for each pixel was set to 0.1 s.

#### Absorption experiment

The absorption measurements were conducted using the same white light laser source as in the dark-field scattering experiment. The light was focused onto the sample through an infinity-corrected objective (Mitutoyo, M Plan Apo NIR 50×) from the bottom of the glass coverslip, and the transmitted light was collected by the same top objective (Mitutoyo, M Plan Apo NIR 10×) and directed through the same optical path as in the dark-field experiment. The excitation power during the absorption experiment was 6 μW, and the exposure time per pixel was 0.1 s.

### SERS measurement and single-particle SECARS measurement

A Renishaw InVia Qontor Raman microscope with 633-nm excitation was used for SERS measurement. A 50× long working distance objective (Leica, catalog no. 566038) was used for single-particle mapping measurements using 633 nm (0.22 mW). To quickly evaluate the SECARS performances and potentials of NPs with different morphologies to achieve single-particle SECARS measurements, we screened diverse NPs via CARS microscope by preparing NP-enriched substrate where NPs were first centrifuged down and then recovered to 10 to 25× of its original concentration. Then, 5 to 10 μl of concentrated NPs was then drop-casted on a glass coverslip and dried in air. For single-particle SECARS measurements, top candidates of NPs with different morphologies that presented good SECARS performance during the screening experiment were first centrifuged down and then recovered by DI water to 5 to 20 times of its original volume (i.e., 5 to 20% of its original concentration). Twenty microliters of diluted NPs was drop-casted on a piece of photoetched glass substrate with 50-μm labeled grids (ibidi, Gridded Glass Coverslips Grid-50) and then slowly blow-dried with nitrogen gas for SECARS imaging. After SECARS imaging, the gridded coverslip was sputtered with a thin layer of Pd/Au. Guided by the grids, SEM imaging was conducted at the exact same ROI as SECARS.

Hyperspectral SECARS imaging was conducted on the same spectral focusing CARS microscope as described by Zong *et al*. (*25*). Briefly, an 80-MHz femtosecond laser (InSight DS+, Spectra-Physics) provided the pump and Stokes beams. For spectral focusing purpose, the pump and Stokes pulses were equally stretched to the picosecond level by five glass rods (SF57, 15 cm per rod) in the common path and one extra rod in the Stokes path. Then, both beams were sent to a laboratory-built laser-scanning microscope with a 60× water immersion objective (NA = 1.2, Olympus) to focus the light on the sample and an oil condenser (NA = 1.4, Olympus) to collect the signals. Signals were obtained by a photomultiplier tube (PMT, H7422-50, Hamamatsu) combined with bandpass filters. To detect the major ν(NO_2_) peak of the 4-NBT indicators, the pump beam was tuned to 912 nm, and the Stokes beam was fixed at 1040 nm. SECARS signals were collected by PMT with an 810/40-nm bandpass filter. For SECARS screening studies where high-density NPs with different morphologies were examined, the power of both beams (pump and Stokes) irradiated on the sample was set to 150 μW. The hyperspectral SECARS imaging stack was collected at 200 × 200 pixels per frame with 120 sequential frames, a pixel size of 1 μm, and a pixel dwell time of 10 μs per pixel per frame. The ensemble SECARS signals of sphere-core SiO_2_-CS NPs collected under the same conditions as screening studies but with slightly higher power set at 200 μW for both pump and Stokes were shown for better data visualization. For single-particle SECARS studies, the hyperspectral SECARS imaging stack with 60 sequential frames was collected at 600 × 600 pixels per frame with a pixel size set at 150 nm and the power of both beams (pump and Stokes) set at 200 μW for sphere-core SiO_2_-CS NPs or at 300 × 300 pixels per frame with a pixel size set at 200 nm and the power of both beams (pump and Stokes) set at 250 μW for star-core CS NPs to cover a whole image area of 90 μm by 90 μm or 60 μm by 60 μm. The imaging was conducted at a speed of 10-μs acquisition time per pixel. All the SECARS spectra were analyzed by ImageJ.

### FDTD simulation and calculation

We further examined the extinction spectrum and distribution of the electromagnetic field excited by 633 nm of star-core CS NPs through the FDTD method using FDTD Solutions (Lumerical Inc.) software. All calculations were performed with a total-field scattering-field source. The empirical optical dielectric data of Au (Johnson and Christy) were fit with Lumerical’s multicoefficient model during the calculations (*63*). The surrounding refractive index of the models was set to 1.33 to mimic an aqueous environment (SERS) or the surrounding refractive index of the models was set to 1.52 to mimic Si surface environment (SECARS). The wavelength range of the optical cross-section simulated corresponds to the UV-Vis experiment, and 200 sampling points were distributed uniformly in this range. The smallest mesh size in the simulation region was 2 nm.

In the CARS process, the incident beams with two different frequencies ω_p_ and ω_s_ interact through the third-order susceptibility [χ^(3)^] of the material, thereby generating a spectrally separated, blue-shifted beam at the anti-Stokes frequency of ω_as_ = 2ω_p_ − ω_s_. Similar to SERS, both excitation (i.e., pump and Stokes) field and the anti–Stokes Raman signal in the CARS process can be boosted by metallic substrates, which are named SECARS. According to classical electromagnetic theory, the scattered fields at the anti-Stokes frequency in SECARS can be expressed as

$$|E_{\text{SECARS}}|\propto \chi^{\left(3\right)}{g_{p}}^{2}g_{s}g_{\text{as}}{|E_{p}|}^{2}|E_{s}|$$ (1)

where χ^(3)^ is the third-order susceptibility of probe molecules; *E*_p_ and *E*_s_ represent the incident pumping and Stokes fields with the respective frequencies of ω_p_ and ω_s_; and *g*_p_, *g*_s_, and *g*_as_ represent the EF of the pumping (*E*_p_), Stokes (*E*_s_), and anti-Stokes (*E*_SECARS_) fields induced by surface plasmonic resonances generated on metallic substrates, respectively. Therefore, the electromagnetic EF for the SECARS signal is given by *G* = *g*_p_^4^*g*_s_^2^*g*_as_^2^, which should be much higher than that for SERS (*G*_SERS_ = *g*_p_^2^*g*_s_^2^) due to its higher-order dependence on incidence power.

To evaluate SECARS enhancements, we first calculate the spatial distributions of near-field amplitude (|*E*/*E*_0_|) of the metallic substrate at three characteristic wavelengths, that is, the pumping laser wavelength (λ = 912 nm), the wavelengths of Stokes (λ = 1040 nm), and anti-Stokes (λ = 812 nm), respectively. Then, based on *G* = *g*_p_^4^*g*_s_^2^*g*_as_^2^, the map of the SECARS EF can be obtained in a plane for different situations.
