# Supplementary material for: Single-particle surface-enhanced coherent anti–Stokes Raman scattering: Nanoparticle design and mechanism
Source: Sci Adv. 2026 Jan 28;12(5):eady0545. doi: 10.1126/sciadv.ady0545 (PMC12851029; doi:10.1126/sciadv.ady0545)
Supplement: Supplementary file 1 — Figs. S1 to S14 [file sciadv.ady0545_sm.pdf]

Science Advances
AAAS

# Supplementary Materials for

## Single-particle surface-enhanced coherent anti–Stokes Raman scattering: Nanoparticle design and mechanism

Sanjun Fan *et al.*

Corresponding author: Sanjun Fan, fan.1113@osu.edu; Ji-Xin Cheng, jxcheng@bu.edu;
Zachary D. Schultz, schultz.133@osu.edu

*Sci. Adv.* **12**, eady0545 (2026)
DOI: 10.1126/sciadv.ady0545

**This PDF file includes:**

Figs. S1 to S14

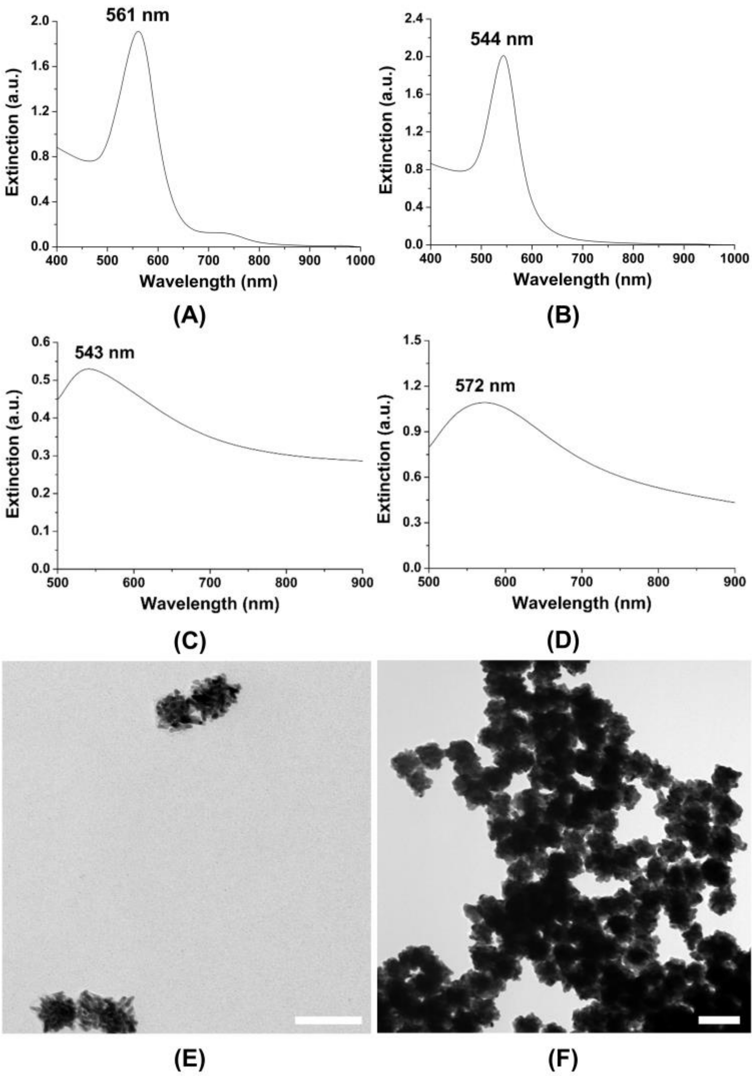


**Fig. S1**. **Extinction spectra and TEM images of nanoparticles.** Extinction spectra of solutions containing heterogeneous Au nanospheres (**A**), from which homogeneous Au nanoparticles (**B**) were synthesized after NaClO & $HAuCl_4$ etching. TEM images of GERTs synthesized using (**E**) 200 μL of 10 mM $HAuCl_4$ solution and using (**F**) 600 μL of 10 mM $HAuCl_4$ solution, respectively. Scale bar: 100 nm. The corresponding extinction spectra of (**E**) and (**F**) are shown in (**C**) and (**D**).

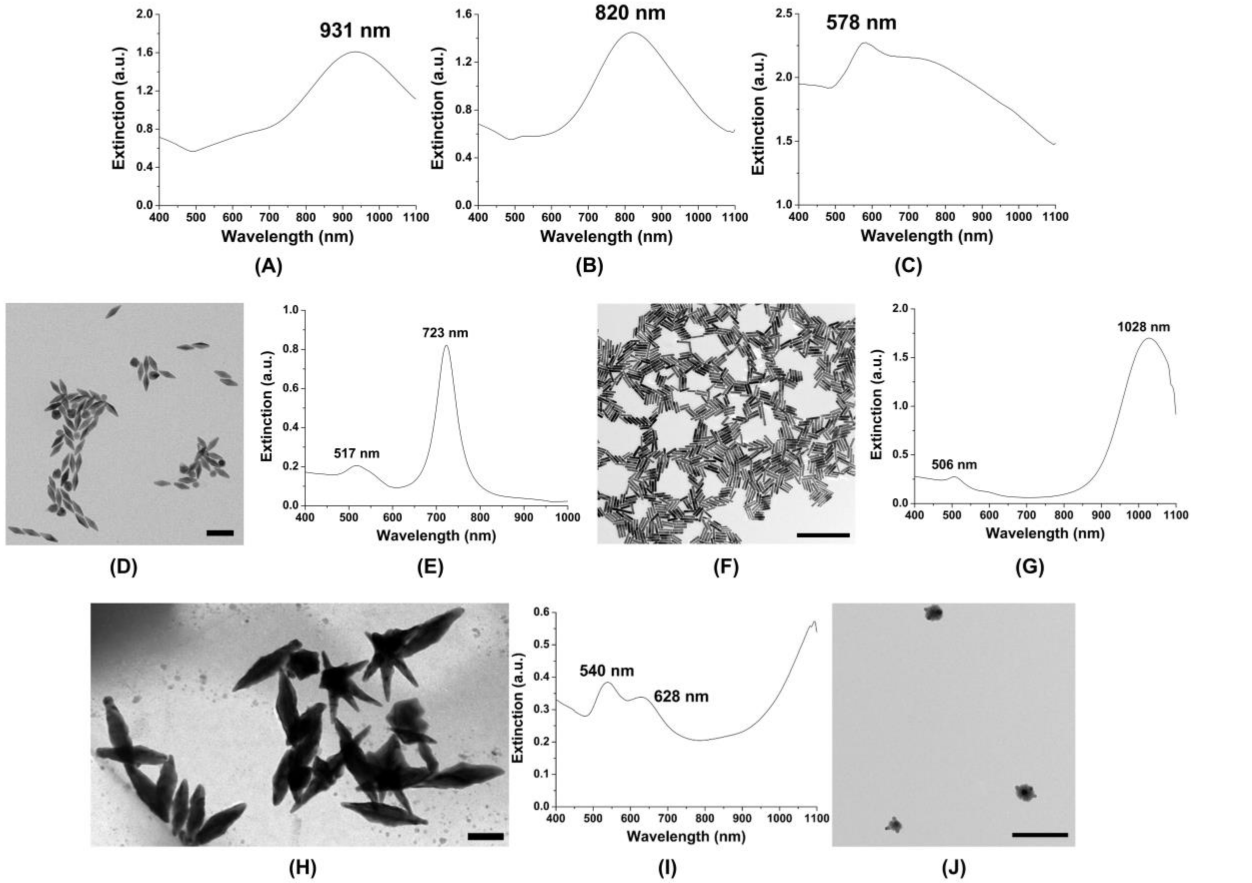


**Fig. S2**. **Extinction spectra and TEM images of nanoparticles.** Extinction spectra of anisotropic Au nanostars (**A**) and (**B**), and isotropic Au nanostars (**C**). (**A**) was synthesized by adding 20 μL of Au seed solution and (**B**) was synthesized by the adding 80 μL of Au seed solution; TEM image of Au bipyramid (6 mL seeds added) (**D**) and its corresponding extinction spectrum (**E**). Scale bar: 100 nm; TEM image of Au nanorod synthesized by adding 1.8 mL of 10 mM $HAuCl_4$ (**F**) and its corresponding extinction spectrum (**G**). Scale bar: 0.5 μm; TEM image of Au bipyramid-based core/shell NP (**H**), and its corresponding extinction spectrum (**I**). Scale bar: 100 nm; Low-magnification TEM image of porous Au star-core CS NPs. The corresponding LSPR peaks were at 520 and 874 nm. Scale bar: 500 nm (**J**).

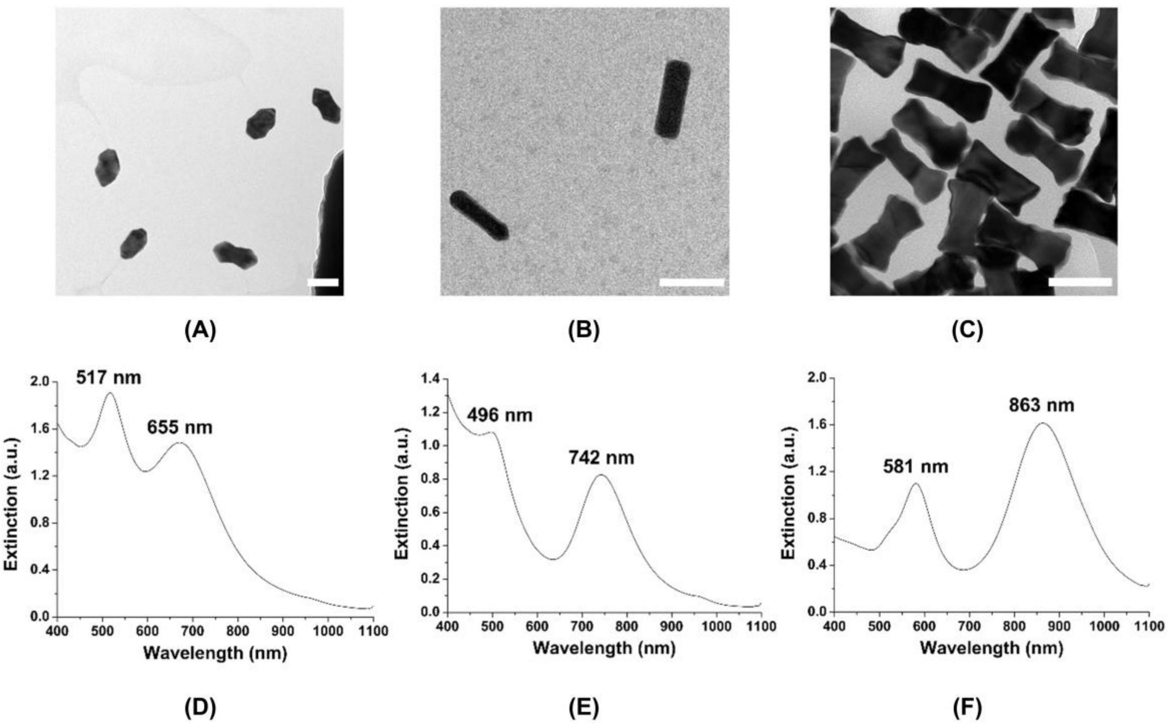


**Fig. S3. Extinction spectra and TEM images of nanoparticles.** TEM images of Au nanorod-based core/shell NPs (**A** (method 1); **B** (method 2); **C** (method 3)). The corresponding extinction spectra of (**A-C**) were shown below in (**D-F**). Scale bar: 100 nm.

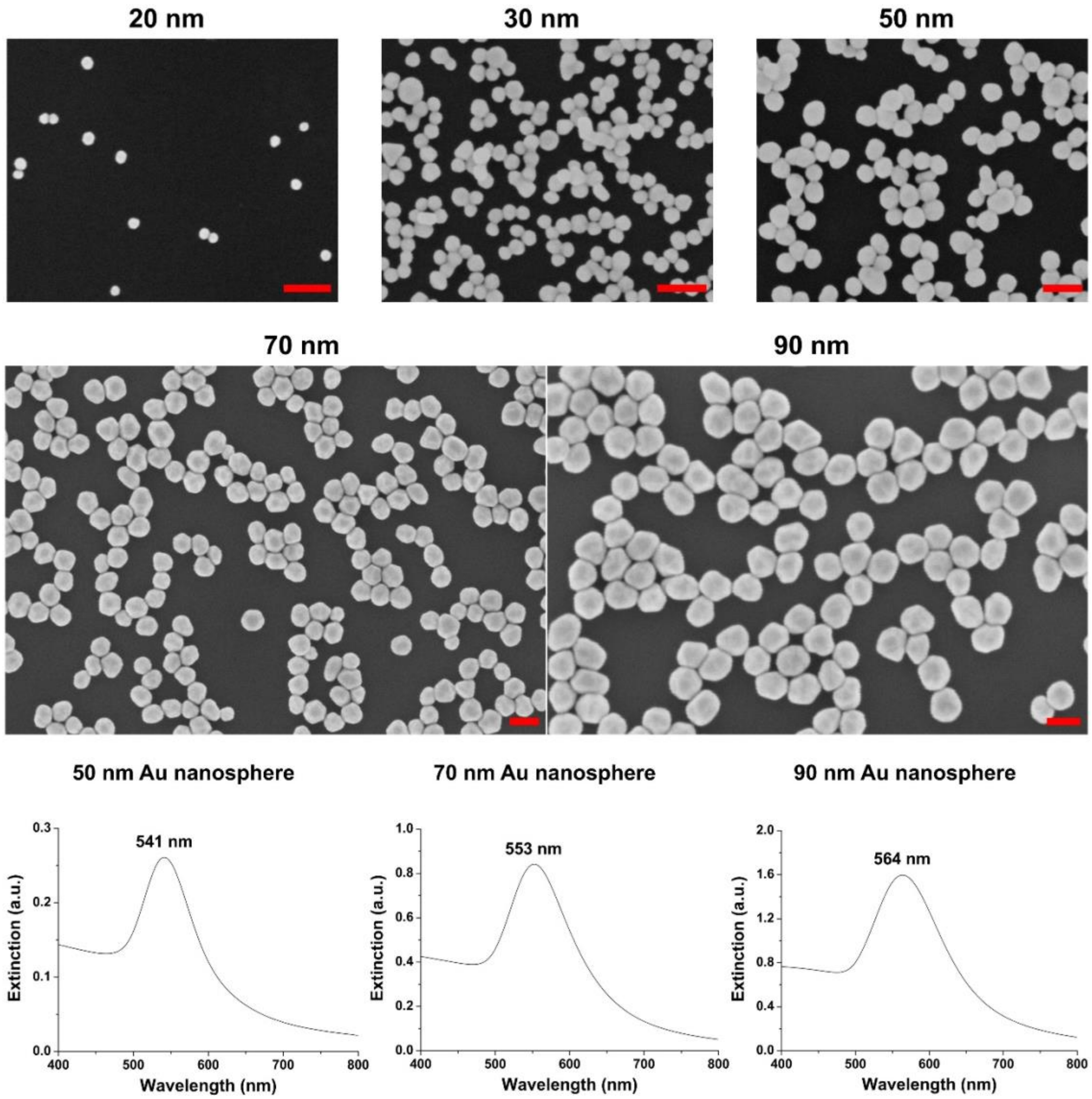


**Fig. S4**. **Extinction spectra and SEM images of nanoparticles.** SEM images of Au NPs (20, 30, 50, 70, 90 nm) used in the preparation of sphere-core CS NPs. The extinction spectra of Au nanospheres (50/70/90 nm) used as cores were shown below. Scale bar: 100 nm.

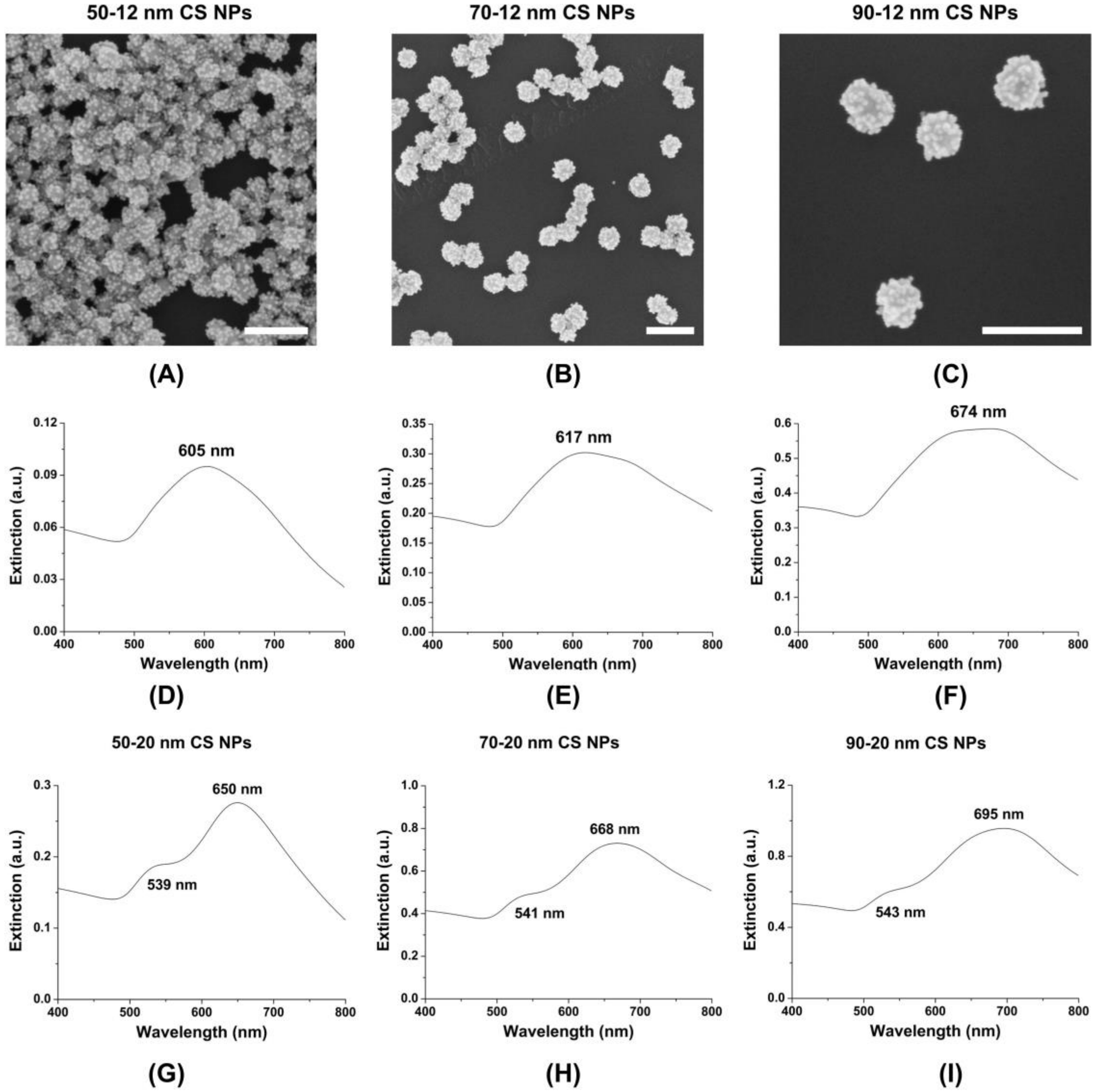


**Fig. S5**. **Extinction spectra and SEM images of nanoparticles.** SEM images (**A-C**) of sphere-core CS NPs made of 50/70/90 nm Au NP cores and 12 nm Au NP satellites before silica shell encapsulation with corresponding extinction spectra shown below (**D-F**). Scale bar: 200 nm; Extinction spectra (**G-I**) of sphere-core CS NPs made of 50/70/90 nm spherical Au NP cores and 20 nm spherical Au NP satellites before silica encapsulation. SEM images were shown in Fig. 2**M-O**.

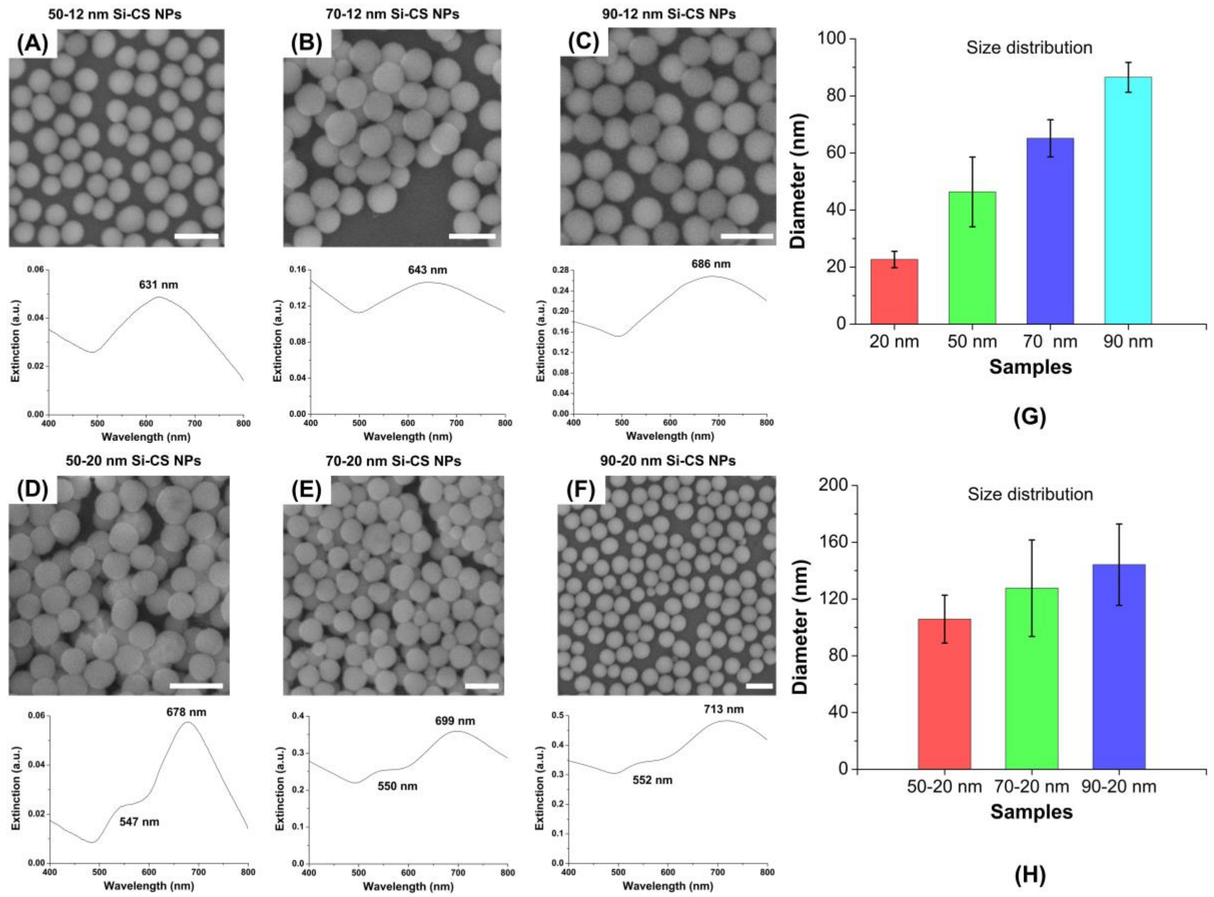


**Fig. S6**. **Extinction spectra and SEM images of nanoparticles, along with their particle size distribution.** SEM images (**A-C**) of silica shell-coated sphere-core CS NPs (sizes of sphere-core CS NPs before encapsulation: 50-12 nm, 70-12 nm, 90-12 nm) with the corresponding extinction spectra shown below. Scale bar: 200 nm; SEM images (**D-F**) of silica shell-coated CS NPs (sizes of core-satellite: 50-20 nm, 70-20 nm, 90-20 nm), and their extinction spectra are shown below. Scale bars are 200 nm; (**G**) Particle size distribution of different nanoparticles (20 nm, 50 nm, 70 nm, 90 nm) collected from various fields; (**H**) Particle size distribution of shell-coated sphere-core CS NPs (50-20 nm, 70-20 nm, 90-20 nm), measured from three different areas to account for sample heterogeneity.

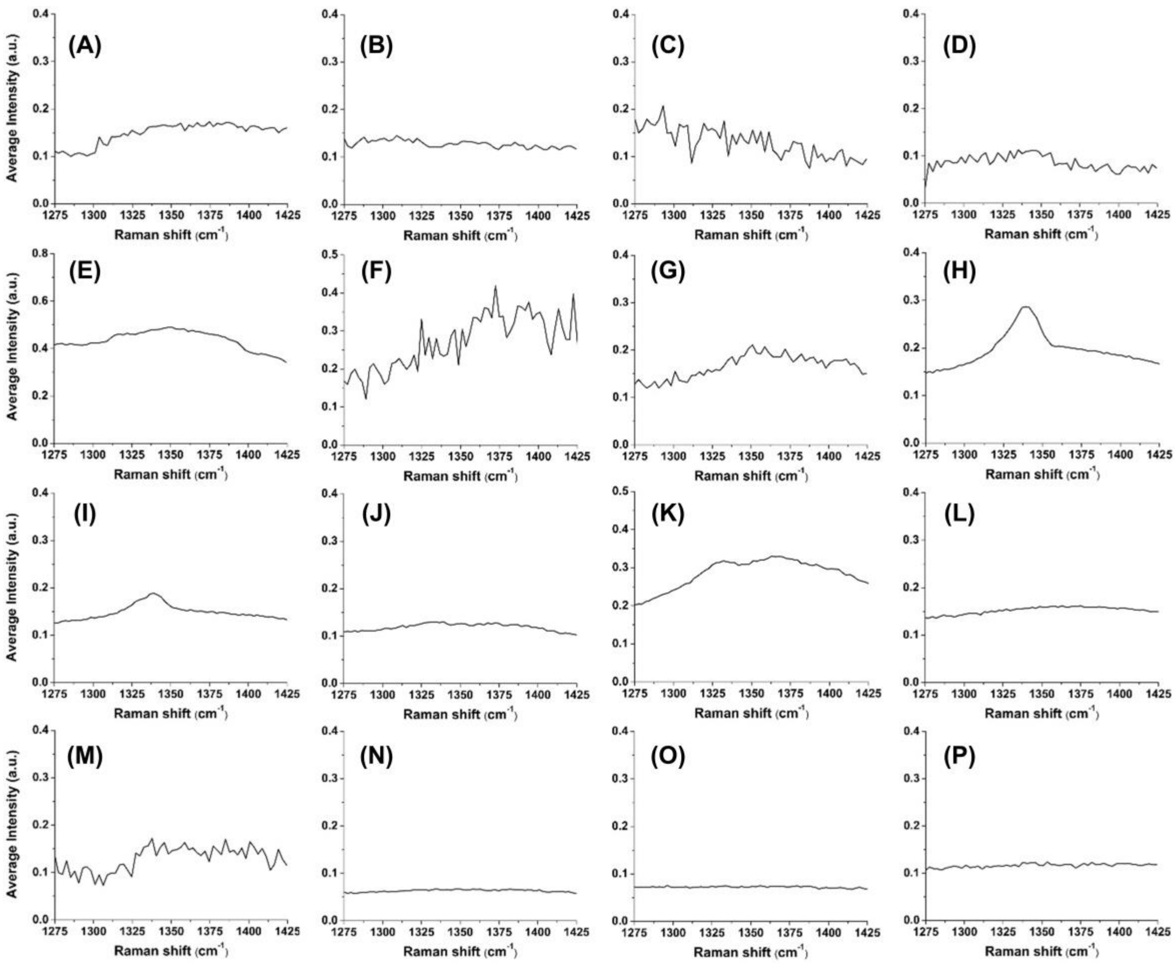


**Fig. S7**. **SECARS screening of nanoparticles with diverse morphologies.** (**A**) Au nanosphere, (**B**) GERTs (200 μL 10 mM $HAuCl_4$), (**C**) Anisotropic nanostars (20 μL Au seed solution), (**D**) Isotropic nanostar, (**E**) Nanorods (0.6 mL 10 mM $HAuCl_4$), (**F**) Nanorod based intra-gap NPs, (**G**) Bipyramid (0.5 mL seed solution), (**H**) Bipyramids based intra-gap NPs, (**I**) Nanostar based porous NPs, (**J**) Core/shell NPs, (**K**) Core/shell NPs, and (**L**) Porous Au star (core)/sphere (satellite) NP, (**M**) Smaller bipyramid (6 mL seeds) in Fig. S2**D**-**E**, (**N**) Au bipyramid-based core/shell NP in Fig. S2**H**-**I**, (**O**) Au nanorod-based core/shell (method 2) in Fig. S3**B**, (**P**) Bigger GERTs (600 μL 10 mM $HAuCl_4$) in Fig. S1**F**.

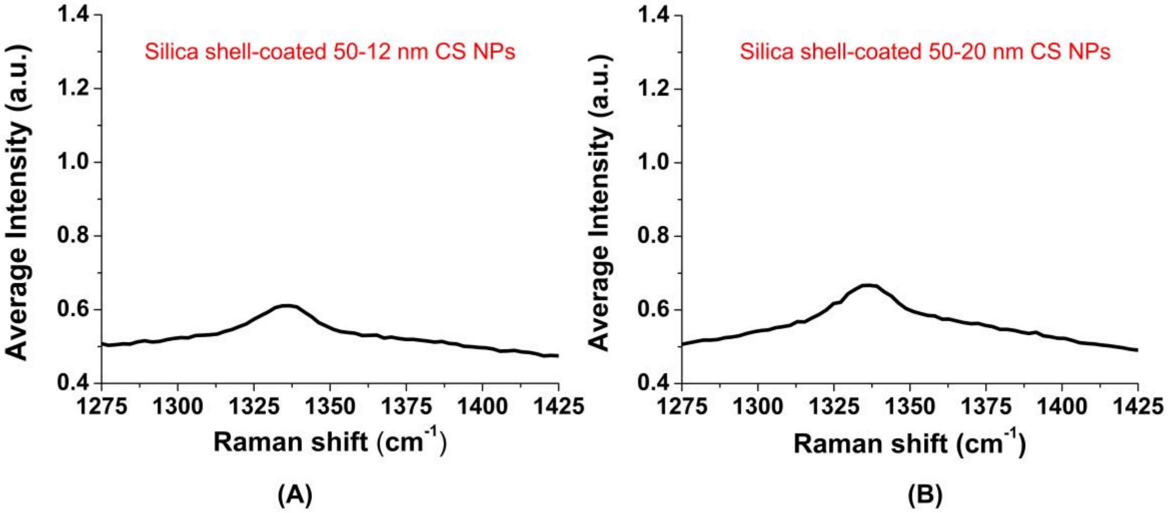


**Fig. S8**. **Average SECARS signals of silica-shell coated sphere-core CS NPs.** (**A**) Silica shell-coated 50-12 nm sphere-core CS NPs; (**B**) Silica shell-coated 50-20 nm sphere-core CS NPs.

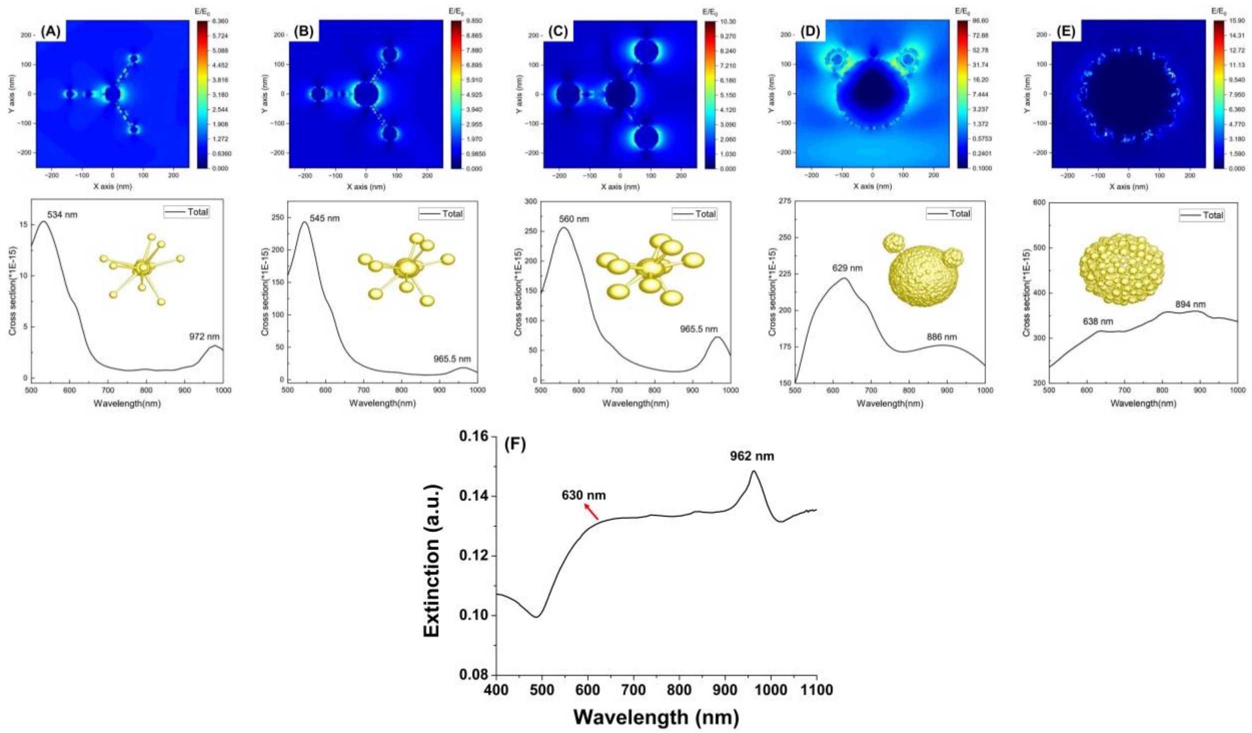

**Fig. S9**. **FDTD simulations of CS NPs, along with their extinction spectra.** FDTD simulations of the electromagnetic field (633 nm) of star-core CS NPs with corresponding logarithmic scale bar on the right to better visualize the results of (**A**) CS NPs (4 mL seed solution, Fig. 4**B**), (**B**) CS NPs (2mL seed solution, Fig. 4**C**), (**C**) CS NPs (1 mL seed solution, Fig. 4**D**), (**D**) CS NPs (0.25 mL seed solution, Fig. 4**F**), (**E**) Spheres (0.125 mL seed solution, Fig. 4**G**) in water. The UV–Vis extinction spectra corresponding to (**A-E**) are shown below. EF of CS NPs (0.5 mL seed solution, Fig. 4**E**) is $31.3 \times 10^4$; Extinction spectrum (**F**) of star-core CS NPs (Fig. 4**F**) in water. Due to some level of heterogeneity of synthetic star-core CS NPs, the experimental UV extinction peaks were not as same as the extinction peaks calculated by simulation that was based on single nanostructure in Fig. S9**D**.

Calculation of experimental enhancement factors based on the method reported in J. Chem. Phys., **2021**, *154*, 034201 and RSC Adv., **2023**, *13*, 27321.

$$EF_{SECARS} = \frac{\frac{I_{SECARS}}{P^2_{pump,SECARS} P_{Stokes,SECARS} N^2_{NP}}}{\frac{I_{CARS}}{P^2_{pump,CARS} P_{Stokes,CARS} N^2_{powder}}}$$

$$P_{pump,SECARS} = P_{Stokes,SECARS} = 0.25mW$$

$$I_{SECARS}\ per\ pixel = \frac{I_{signal-background}}{Pixels\ per\ NP}$$

$$P_{pump,CARS} = 18mW, P_{Stokes,SECARS} = 1mW,$$

$$I_{CARS}\ per\ pixel = 10.19,\ N_{powder} = 4.9 * 10^9$$

$$EF_{SECARS} = \frac{\frac{\frac{I_{signal-background}}{Pixels\ per\ NP}}{0.25^2 * 0.25 * N^2_{NP}}}{\frac{10.19377333}{18^2 * 1 * (4.9 * 10^9)^2}} = \frac{\frac{I_{signal-background}}{Pixels\ per\ NP}}{N^2_{NP}} * 4.884 * 10^{22}$$

$N_{NP}$ is the number of 4-NBT molecules and the topological polar surface area of 4-NBT is 0.468 $nm^2$.

Estimated surface area of Fig. 4**F** = 287,388.5 $nm^2$; Estimated surface area of Fig. 4**D** = 226,406.5 $nm^2$.

$N_{NP}$ = S/0.468 $nm^2$.

According to the equation above, EFs of CS NPs (Fig. 4**D**) and (Fig. 4**F**) are from $8.9 \times 10^9$ to $3.6 \times 10^{11}$ and from $2.3 \times 10^{10}$ to $3.1 \times 10^{11}$, respectively.

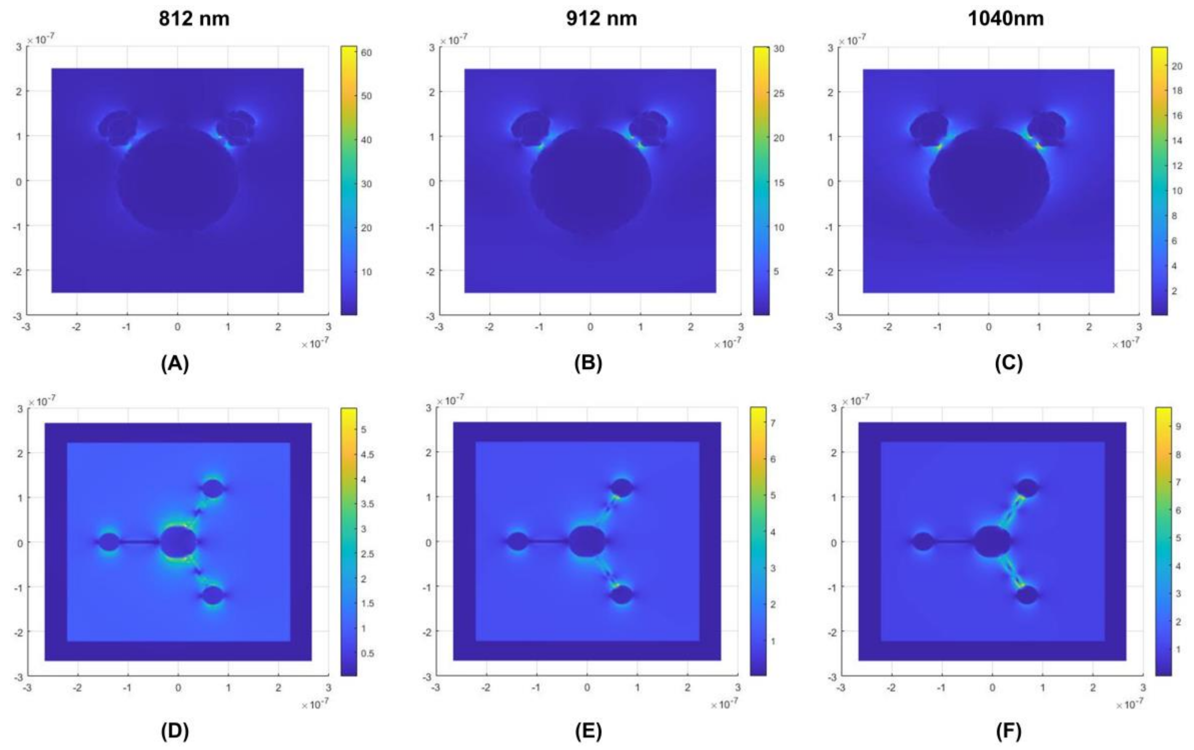


**Fig. S10**. **FDTD simulations of CS NPs.** FDTD simulation of the field enhancements at SECARS anti-Stokes/pump/Stokes wavelengths (812/912/1040 nm). (**A-C**) star-core CS NPs (Fig. 4**F**), (**D-F**) star-core CS NPs (Fig. 4**C**).

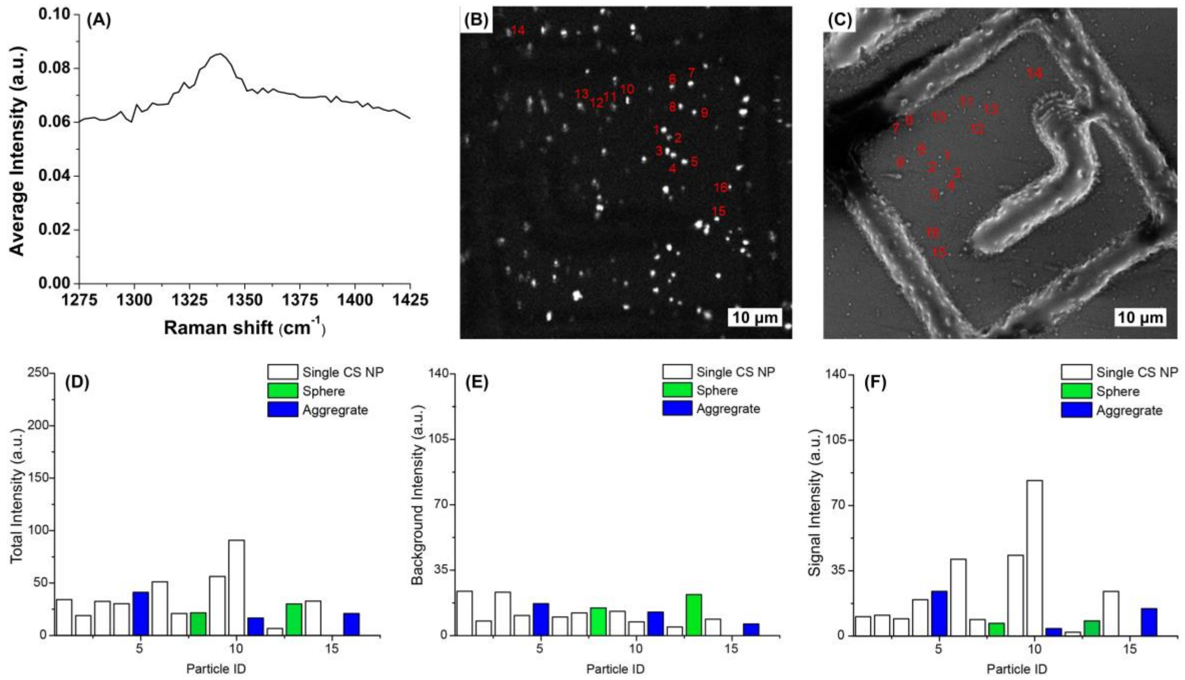


**Fig. S11**. **Single-particle SECARS of core-satellite NPs (Fig. 4D).** (**A**) Averaged SECARS spectrum of the whole ROI as shown in (**B**) with randomly dispersed star-core CS NPs (1 mL seed solution, Fig. 4**D**), (**B**) The averaged SECARS image of the hyperspectral imaging of a randomly selected ROI, (**C**) SEM image of the photoetched glass substrate with labelled grids with the same ROI, (**D**) Total intensity of NPs, (**E**) Background intensity of NPs, (**F**) Signal intensity (total intensity – background intensity) of NPs. Particle ID corresponds to the numbers in (**B** or **C**), NP 15 presented the sign of photodamage and therefore excluded.

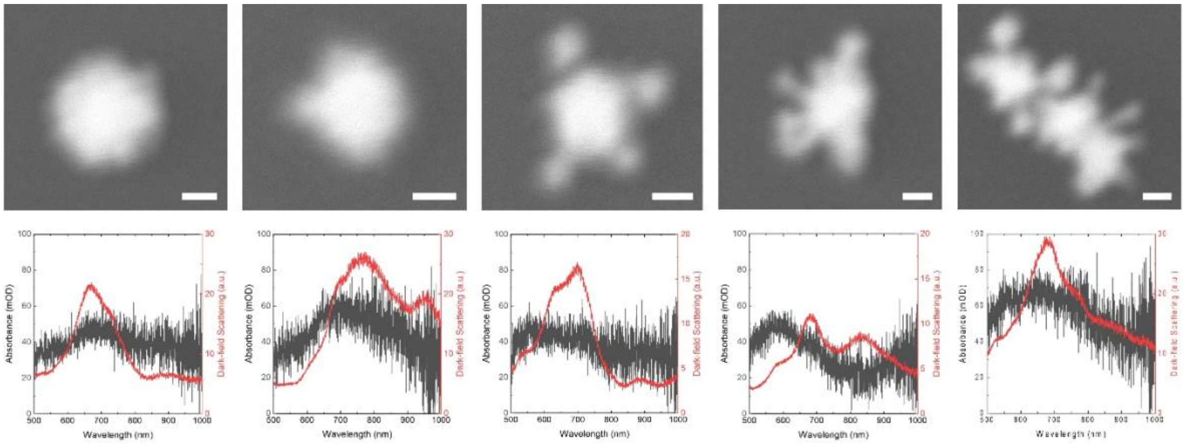


**Fig. S12**. **SEM images of CS NPs (Fig. 4F), along with their extinction spectra at the single-particle level.** The representative NPs with different morphologies existed in synthetic star-core CS NPs (Fig. 4**F**), including spheres, star-core CS NPs, and aggregates were selected as shown in SEM images with their corresponding single-particle/single-cluster extinction spectra (absorbance: black and dark-field scattering: red) on glass shown as below.

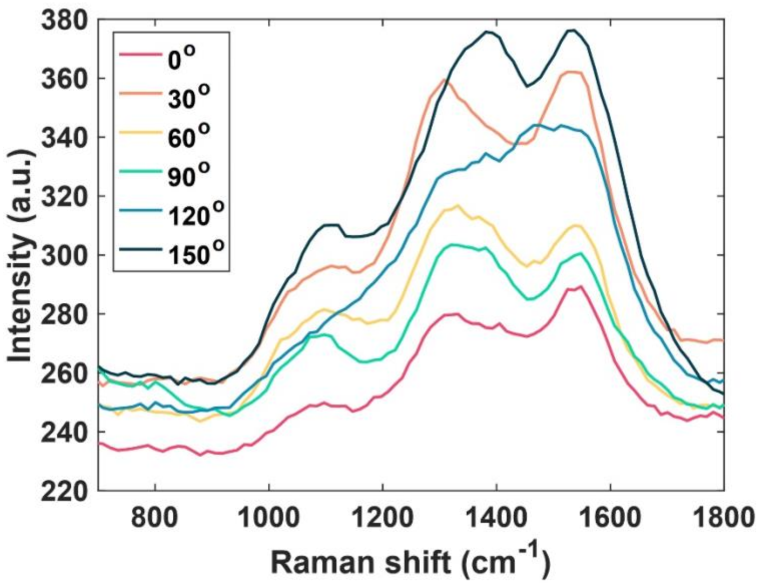


**Fig. S13**. **SERS spectra of CS NPs.** SERS spectra of single star-core CS NP at different polarization angles changing from 0° to 150°.

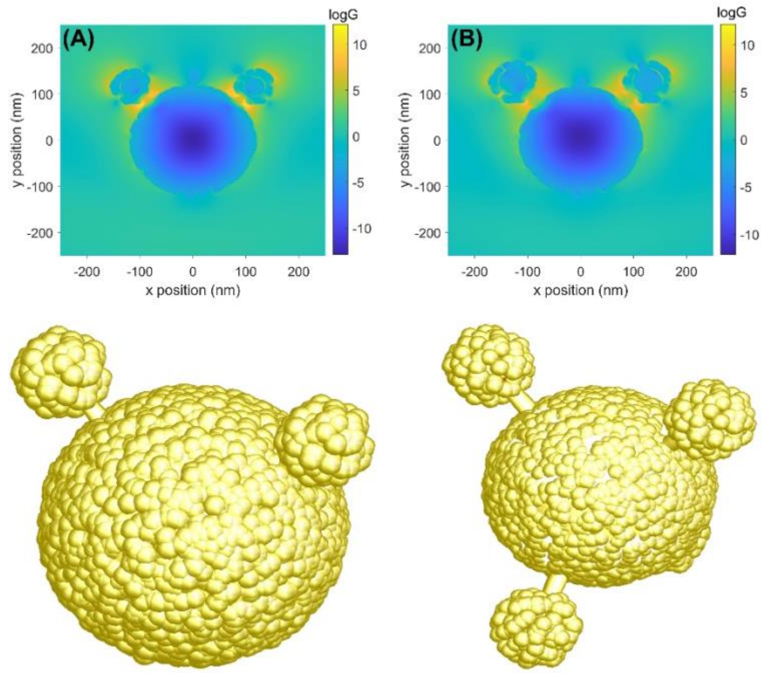


**Fig. S14**. **FDTD simulations of asymmetric and symmetric CS NPs.** The total enhancement factors ($G= g_p^4 g_s^2 g_{as}^2$, $g_p$, $g_s$, $g_{as}$: enhancement factor at the wavelengths of pumping (912 nm), Stokes (1040 nm) and anti-Stokes (812 nm)) are presented for (**A**) Asymmetric CS NP (Fig. 3**F**, EF = $10^{13}$) and (B) Symmetric CS NP (Fig. 3**F**, $10^{12}$) on glass. Their corresponding 3D maps were shown below.
